# Application of green pomelo peel essential oil-based carboxymethylcellulose coatings reinforced with nano chitosan and nano cellulose fibers during the drying process on dried silkworms

**DOI:** 10.1038/s41598-025-93243-7

**Published:** 2025-03-13

**Authors:** Tran Thi Van, Fumina Tanaka, Meng Fanze, Mohammad Hamayoon Wardak, Dong Thanh Pham, Ata Aditya Wardana, Laras Putri Wigati, Xirui Yan, Fumihiko Tanaka

**Affiliations:** 1 Graduate School of Bioresource and Bioenvironmental Sciences, Kyushu University, 744, Motooka, Nishi-ku, Fukuoka-shi, Fukuoka, 819-0395 Japan; 2 Department of Agri-food Preservation Technology, Vietnam Institution of Agricultural Engineering and Postharvest Technology, Ha Noi, 10000 Vietnam; 3 Faculty of Agriculture, Kyushu University, W5-874, 744, Motooka, Nishi-ku, Fukuoka-shi, Fukuoka, 819-0395 Japan; 4 Department of Food Technology, Faculty of Engineering, Bina Nusantara University, Jakarta, 11480 Indonesia

**Keywords:** Green pomelo peel essential oil, Nano chitosan, Drying process, Silkworm, Metabolite process, Environmental sciences, Chemistry, Materials science, Nanoscience and technology

## Abstract

The application of edible coatings to dried products such as silkworms (*Bombyx mori*) is an innovative method for preserving nutrients during drying process. These coatings are made from safe, natural ingredients such as carboxymethylcellulose (CMC), nano cellulose fibre (NCF), nano chitosan (N-Ch), and varying concentrations of green pomelo peel essential oil (GPO). The results revealed that the addition of GPO into coating/film significantly changed the pH, viscosity, and TSS values of the coating solutions while reducing film weight loss, particularly in the CCG3 film (61.93%). CCG3 also showed the strongest antibacterial effect against “*Staphylococcus aureus*” and maintained lower silkworm moisture content compared to other samples. Metabolic analysis revealed that certain compounds, such as 2-Hydroxyglutaric acid-3TMS and malic acid-3TMS, were more robust in CCG3. X-ray CT image analysis using image processing technology showed high accuracy, with the porosity area in the CCG3 sample being greater. These findings highlighted the potential application of edible coating in drying process to protect the nutrients of dried products.

**Supplementary Information:**

The online version contains supplementary material available at 10.1038/s41598-025-93243-7.

## Introduction

Edible insects have been studied for their potential as a food source providing essential nutrients for humans^1^. Silkworm pupae are the main byproduct after the process of spinning silk threads on cocoons. Silkworm provides a high protein source (48.31–67.39%) including all essential amino acids (0.17 – 3.92%)^2^. Drying silkworm helps prolong the shelf life of preservation which is an intermediate step for other uses. However, the drying condition might alter compounds, or change the sensory properties of the dried product^3^. For example, drying methods differentially altered the main volatile components of edible grasshoppers and silkworms, including the change in appearance, flavor, texture. The change rate varies depending on each drying method^3^. To prevent the nutritional value of dried silkworm, a convenient and suitable drying method is a requirement for scientific research nowadays. Besides, a novelty method which could assist silkworm drying process is also needed. One of recommendations is that the use of edible coating to cover the surface of silkworms and protect them from the heat of the drying process. The mechanism of the covering is creating a thin film on the sample surface to protect samples from unshielded contact with heat or airflow within the drying system. In this study, the purpose of the coating is to cover the surface of silkworm before drying process so it can prevent samples from heat and airflow during drying, thereby minimizing damage to surface texture, appearance, retaining the most nutritional compounds of dried products.

The environmental and human health problems associated with petroleum-based synthetic plastic materials used in the food sector were attracting the attention of scientists and people around the world. Choosing coatings from natural and edible materials is a priority in the current time. Nanomaterials show better improved mechanical properties such as gas barrier properties, improved dimensional stability, and better thermal expansion^4^. Nano-chitosan (N-Ch) is a particle with a small molecular size of a few hundred nm. The roles of N-Ch were to provide excellent properties that improve the functional characteristics, and strengthen the chitosan film, it also have a positive effect on the antibacterial activity of the membrane^5^. To perform its role as filler in the membrane complex, N-Ch must not be dissolved in the system, and the protonation of weakly basic amine groups requires the pH of the coating solution to be maintained at a higher level than its pKa (pKa ≈ 6.3–6.5)^6^. Through carboxymethylation, sodium carboxymethyl cellulose was produce^7^. This modified cellulose can soluble in water, offers advantageous properties in film-forming, and provides moderate air penetrability^8^. Due to its robust internal network structure, this material is considered an active hydrogel, contributing to enhanced composite membrane performance^7^. Combination of natural oils or nanomaterials in CMC based could alter the water-resistant characteristics of CMC films and improve their mechanical and antibacterial properties^9,10^.

Essential oil in general plays an important role in both antibacterial, antioxidant and film-forming dispersions, especially in essential oil concentration^11,12^. These authors also mentioned that the nature or higher concentration of essential oils such as tea tree, bergamot, and lemon essential oils into chitosan or hydroxypropyl methylcellulose could lead to higher antibacterial effect and film physico-chemical characteristic changes such as the lower sorption of moisture or the water vapor permeability. However, these are some authors who were concerned about the negative effects of the essential oils on food properties such as sensory properties and consumer acceptance^13^. Therefore, the selection of essential oil combining with edible coatings also plays a crucial role in the application. In this study, green pomelo peel essential oil (GPO) was selected because of its regional plant area specificity, which was extracted from green pomelo peel in Ben Tre province, Vietnam. The main components of GPO were confirmed containing limonene, α-pinene, β-myrcene and sabinene^14^, those components play an important role in antimicrobial contamination and maintaining the quality of agricultural products^15^.

The use of deep learning techniques, such as instance segmentation algorithms, is increasingly prevalent in agriculture. These algorithms, like YOLO, identify and classify objects in images by assigning class labels, unique instance IDs, and pixel-wise masks, providing detailed object information^16^. YOLOv8, an improvement over YOLOv5, advances in structural design, loss computation, data augmentation, training strategies, and inference processes, making it highly versatile across visual domains^17^. For instance, combining YOLOv8 with an enhanced DeepLabv3 + achieves 90.8% mean intersection over union in plant leaf segmentation^18^, while its application with CSP and C2f modules reaches 99.5% accuracy in classifying fruit ripeness^19^. Previous studies revealed that YOLOv8 was applied to detect multi-stage apple fruit in farm^20^ or fruit freshness^21^ with high accuracy. Besides, X-ray CT is a useful non-destructive technique which was applied to detect the change of internal structure of persimmon and strawberry^22,23^. This study used X-ray CT imaging and deep learning to analyze porosity differences in the inner structure of silkworms before and after drying, comparing coated and uncoated samples to find out the effect of treatments.

In previous studies, we determined the effect of CMC/NCF combined with mandarin peel extraction and/or 1-MCP. The results revealed that the addition of mandarin peel extraction or 1-MCP plays a vital role to control strawberry weight loss, reduce the fungal growth, and significant changes in color and average roughness values of the film. However, there were still some disadvantages such as the increase of the peel extraction concentration did not show the higher effect on prolonging the shelf life of strawberries or the dilution method of 1-MCP might increase the concern about human health^24,25^. From our perspectives, there were no reports which determined the effect of edible coatings on drying process, especially on dried silkworms which must suffer a heat treatment until now. Therefore, this study aimed to combine GPO based on CMC and N-Ch/NCF without any emulsifiers into the edible coating to clarify the effect of this oil on bacterial resistance, film forming, and drying process, to check whether it has any effect to prevent the quality change on silkworm under heated condition.

## Results

### Coating and film characteristics

#### Physical characteristics

pH parameters (Table S1) range from 9.02 to 9.30, the solutions have similar pH, in which CCG1 solution has the highest pH of 9.30. The pH value of the solution depends on the components present such as CMC, CNF, N-CH and GPO, where the pH value of GPO is 3.43. The pH value was confirmed that it affects to mechanical characteristics, color, antibacterial, and antioxidant of edible films^26^.

One of the important parameters that affects the film thickness forms on a food product, is the viscosity of coating solution. In this study, the coating solution viscosity ranges from 141.77 to 167.90 mPas (Table S1). The lowest viscosity value was 141.77 observed for coating solutions including CMC/CNF/N-CH, while viscosity increased for coating solutions with GPO added. Our pervious researches also represented the same the results^15,27^. Previous studies explained that the increase in viscosity upon the addition of essential oils to edible coating solution might be due to molecular interactions between essential oils and polymer matrix. Because essential oils in general or GPO are complex mixtures of hydrophobic compounds such as terpenes, phenols, and alcohols that can interact with the polymer matrix of the edible coating such as polysaccharides, proteins, or lipids. These interactions can lead to the formation of hydrogen bonds, or hydrophobic interactions, which increase the overall resistance to flow, thereby increasing viscosity^28^. Other reasons might be that essential oils have hydrophobic nature, and their incorporation into aqueous-based edible coatings can cause phase separation or aggregation. The hydrophobic interactions between oil molecules and the polymer chains can create a more rigid network, further increasing viscosity^29^. In addition, the addition of essential oils can restrict the movement of polymer chains in the coating solution, leading to increased viscosity^30^.

The average film thickness (Table S1) was 0.050–0.064 mm in this study. The incorporation of different materials which added to films might affect to the thickness of the films^24^. The current results showed that the highest thickness value was found in films CCG3 which was made of CMC/CNF/N-CH and the highest concentrations of GPO, while the lowest value was found in the film with the lowest concentration of GPO (*P* > 0.05).

The nature of GPO is colorless, and it has a light smell of pomelo peel. After being analyzed, the results revealed that there were no significant differences among the color of films (Table S1). This might be the results of the film-based materials and GPO. Some studies demonstrated that edible coatings changed the color of the stored objects such as mandarin extract coatings affected the color of stored strawberry^15^, pectin-based coatings affected tomato color during low temperature storage^31^.

#### SEM

**Fig. 1:**
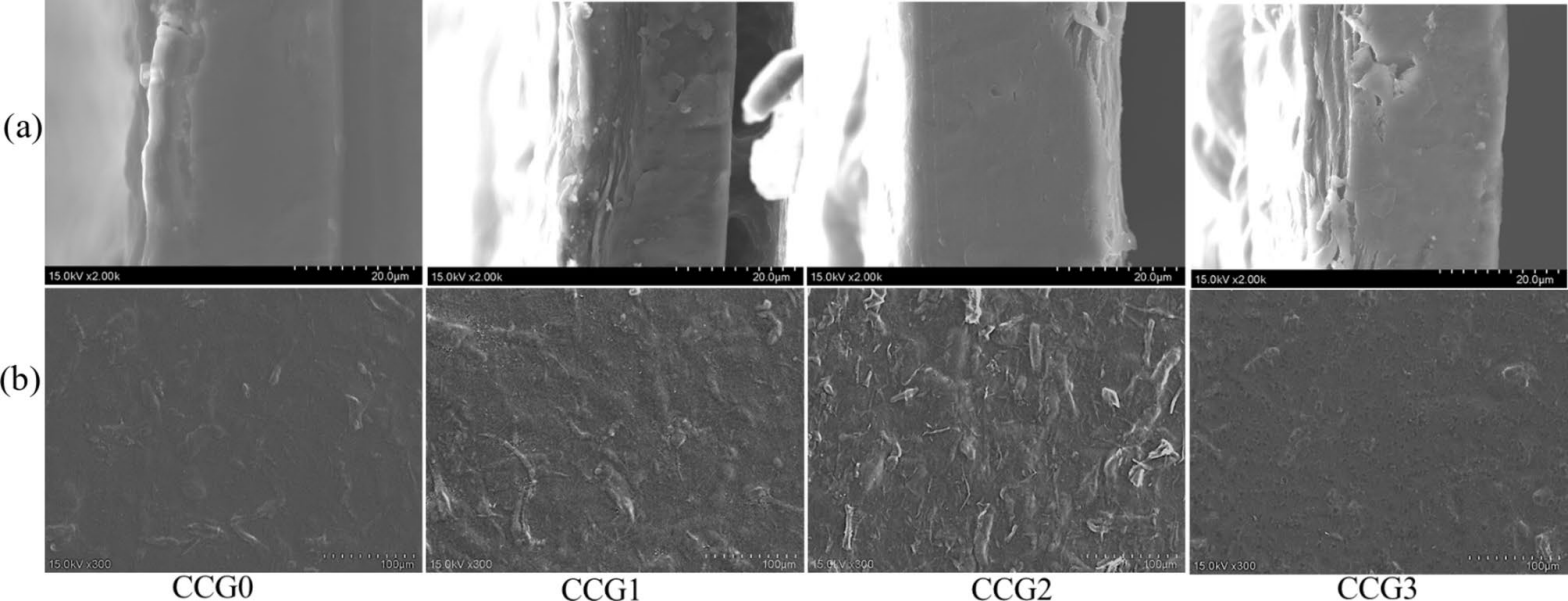
Cross-section (**a**) and surface (**b**) SEM images of the films with and without GPO.

The cross-section and surface structure of the films were illustrated in Fig. 1. According to Ghosh and Katiyar^32^, the film’s properties are directly influenced by the interactions among the components involved in film formation. The surface features of films based on CMC/CNF exhibited irregularities and varied textures due to gelatinization. Films containing GPO (CCG1, CCG2, and CCG3) displayed porous structures with numerous voids distributed throughout. Furthermore, an increase in GPO concentration resulted in greater heterogeneity and increased porosity in the film, leading to noticeable alterations in shape and droplet size within the film. Incorporations of GPO into the film composite replaced robust interactions among polymer networks with lesser interactions between the polymer composite and GPO. This substitution ultimately led to a film with reduced integrity, as evidenced by the presented images. This situation might due to the distribution between hydrophilic and hydrophobic layer in the film matrix^33^. Similar outcomes in films produced were observed from CMC and Ajowan essential oil, noting that an elevated concentration of Ajowan essential oil resulted in a heterogeneous and less cohesive film^34^. Furthermore, Zhang et al.^35^ confirmed the effect of essential oils on polysaccharide base film of sodium alginate matrix with holes were detected on surface and ellipse microspores on cross-section of SEM. The author argued that lipid aggregation might lead to this phenomenon.

#### Atomic force microscopy

**Fig. 2:**
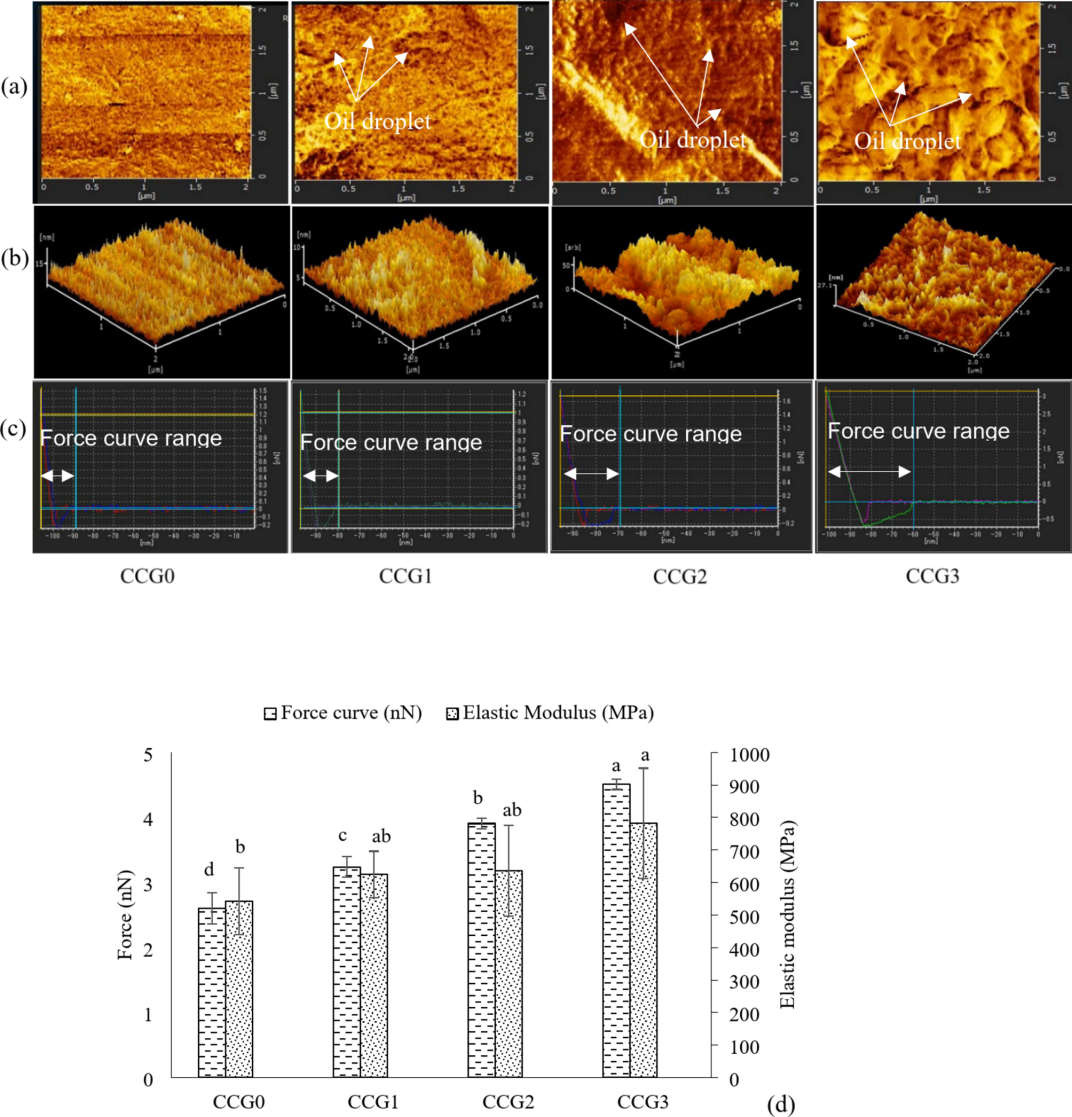
AFM visual images (**a**) – (2D), (**b**) – (3D), (**c**) – force curve visual, and (**d**) - force curve analysis.

The characteristics of the film layers were elucidated through AFM imaging, depicted in Fig. 2a, b, with corresponding results of force curve and elastic modulus indices presented in Fig. 2c, d. From the images, the CCG0 films showed uniform morphology. Conversely, an increase in GPO concentration, particularly in the CCG2 and CCG3 films, led to a substantial increase in inhomogeneous film surface. The film structure was affected by the adding of the oils in the matrix which was confirmed by a lots of studies^15,25,35,36^ which explained that the ratio of saturated fatty acid and unsaturated fatty acid in edible oil also affected to the film surface characteristics. Wongphan et al.^37^ explained that the size and numbers of the particles reduced when adding essential oil in the film matrix, leading to the change in film surface properties. The elevated roughness values might indicate the impact of oil concentrations in the coating film and the dispersion of coating components during homogenization, casting, and drying processes.

The AFM system captures surface images and evaluates nanoscale material properties, assessing film adhesion through force curves. Adhesion force is measured during probe retraction by the maximum attractive force separating the probe from the film surface. The difference among force curves depend on the material properties and film surface characteristics, which represent by the distance between the surface of samples and cantilever probe, it also reflects how harness the film was^38^. Additionally, the elastic modulus reflects a material’s resistance to elastic deformation, adhesion signifies the pull-off force necessary for the cantilever to overcome adhesive interactions between the tip and the sample, dissipation measures the energy dissipated during tip-surface interaction, and deformation encompasses both elastic and plastic deformation^39^. In this study, results indicated that the force curve and elastic modulus increase significantly (*P* < 0.05) with films contain GPO such as CCG1, CCG2 and CCG3 compared to CCG0 (without GPO). The force curve and elastic modulus values of CCG3 were highest compared to other films (*P* < 0.05). A study on the role of sunflower oil in hydroxypropyl methylcellulose based oleogels in chocolate indicated that the harder texture need the greater force to penetrate on it^40^. The increase in force curve values indicated the changes in the molecular structure among film samples^38^, where the concentration of lighter molecules such as aroma or oil components increased. In addition, Yin et al.^36^ explained that the oil with high saturated fatty acid content led to reduce the cutting force. In this study, the force curve of films increases along with the rise of oil concentration, this might suggest that GPO contains more higher unsaturated fatty acids than saturated fatty acids. The increasing of elastic modulus values might indicate that the films without GPO were stiffer than the films contained GPO. These results were in accordance with the appearances of 2D and 3D AFM images which represented the effects of GPO on surface and cross-section of the films.

#### Thermal analysis

**Fig. 3:**
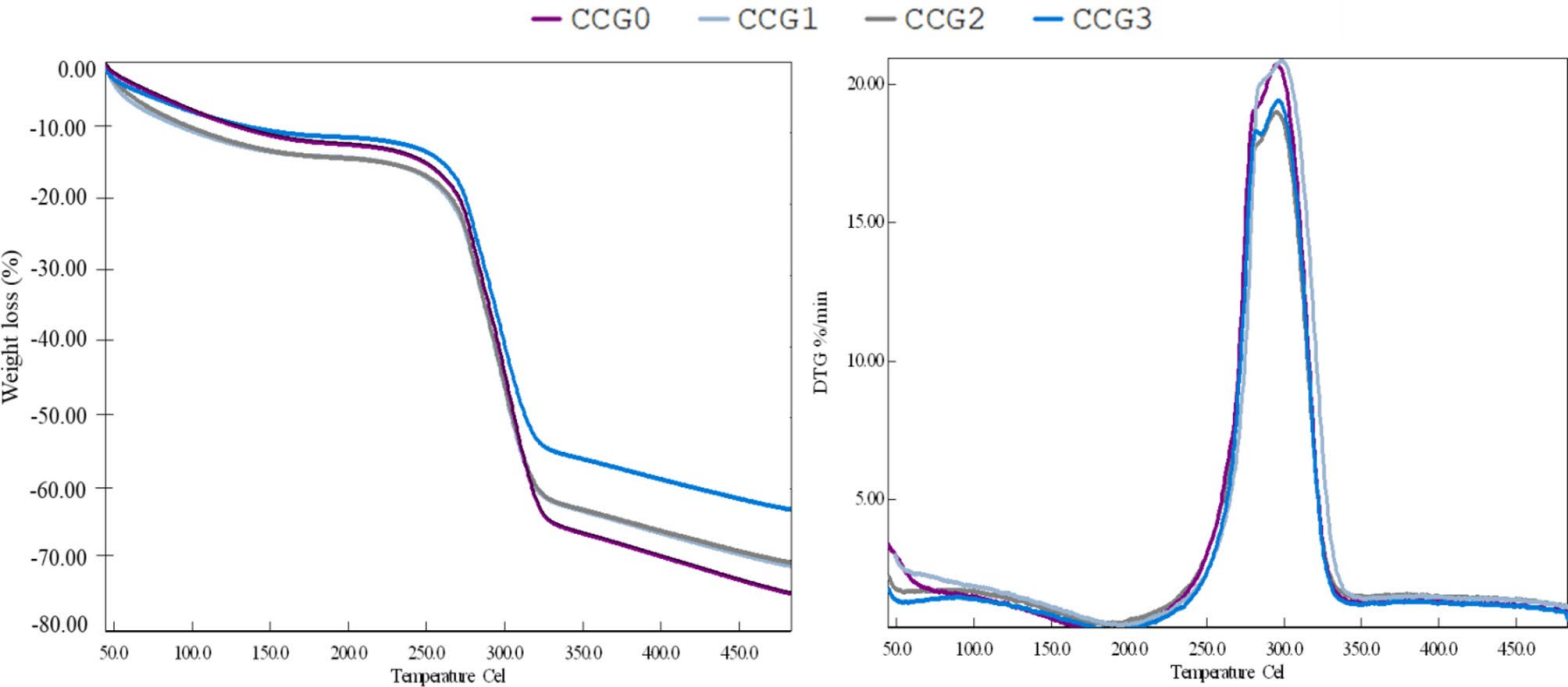
Thermal analysis TG and DTG curves of cellulose edible films.

The thermal properties of films were shown in Fig. 3 and Table S2 which indicated the weight loss of all films dramatically increases following by the temperature rise. The results indicated that the weight loss process was divided into three steps following by a light difference in thermal temperature of each film. Firstly, the temperature rose from 45 °C to 246 °C, which was dominantly due to the loss of free and bound water as well as the loss of their molecular weights through evaporation, or volatile components in materials^35^. Secondly, the range varied at 246 –328 °C. In this step, the weight loss significantly increased, this might cause from the thermal activity associated with natural CMC/CNF/N-CH base, which were main content of the film matrix. At this point, the hydrogen bond chain is broken with the evaporation of glycerol and polyoxyethylene (20) sorbitan, and the sugar in the polysaccharide is broken down. Finally, the temperature was observed from 328 °C to 486 °C, which related to the monosaccharides such as glucose, mannose with decomposition of the carbon residue produced in the second step. After finishing the heating process, the weight loss values of CCG0, CCG1, CCG2, and CCG3 were 74.6%, 70.88%, 70.35%, and 61.93%, respectively, which represented that the value of CCG0 was highest while CCG3 was lowest. The causes might be the cross-linking reaction among materials in the film matrix^41^. This result suggested that the CCG3 film might have the highest stability against the heating process.

### Antimicrobial activity

**Fig. 4:**
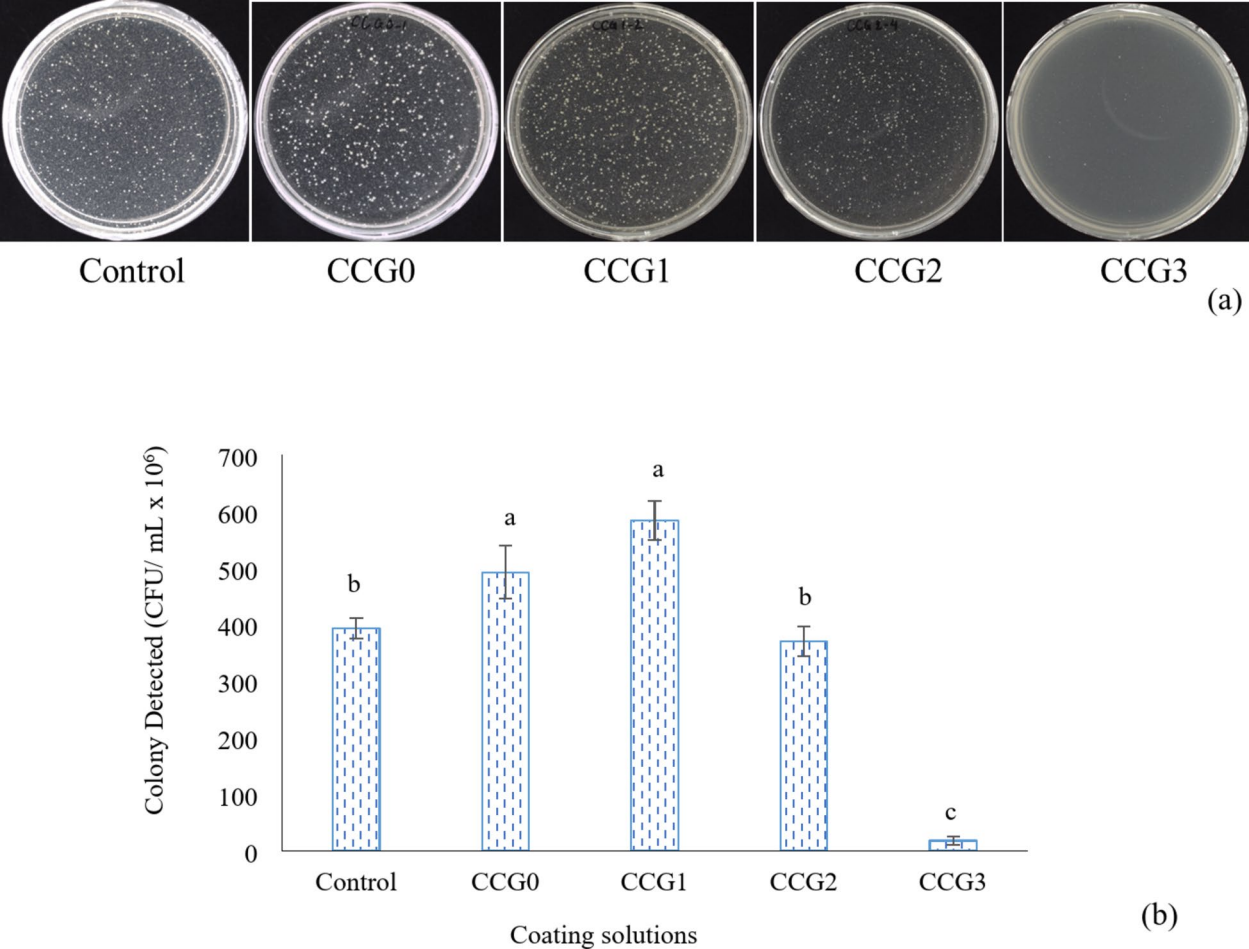
Antibacterial of edible coatings (**a**) – visual recognition, (**b**) – colony analysis.

The antibacterial efficacy of edible coatings derived from CMC/CNF/GPO after 24 h of cultivation is illustrated in Fig. 4a and b. After one day of cultivation at 37 ^o^C, the inhibition effect of Control and CCG2 samples was higher than the CCG0 and CCG1 (*P* < 0.05) while CCG3 exhibited the highest inhibitory effect on *L. aureus* compared to other groups (*P* < 0.05). The reason why the inhibition effect on *L. aureus* of CCG0 and CCG1 was lower than the other group might be from the concentration of GPO in CCG0 and CCG1, which was negative or not enough to inhibit the growth of bacteria in their optimism environment. Similar observations have been reported from incorporating cinnamon essential oil and CMC (Fattahi et al., 2020). These findings align with previous studies, mandarin EO against *E. coli ATCC 25,922*^42^ and *P. digitatum* and *P. italicum*^43^.

### Silkworm pupae application

#### Moisture content of silkworms

**Fig. 5:**
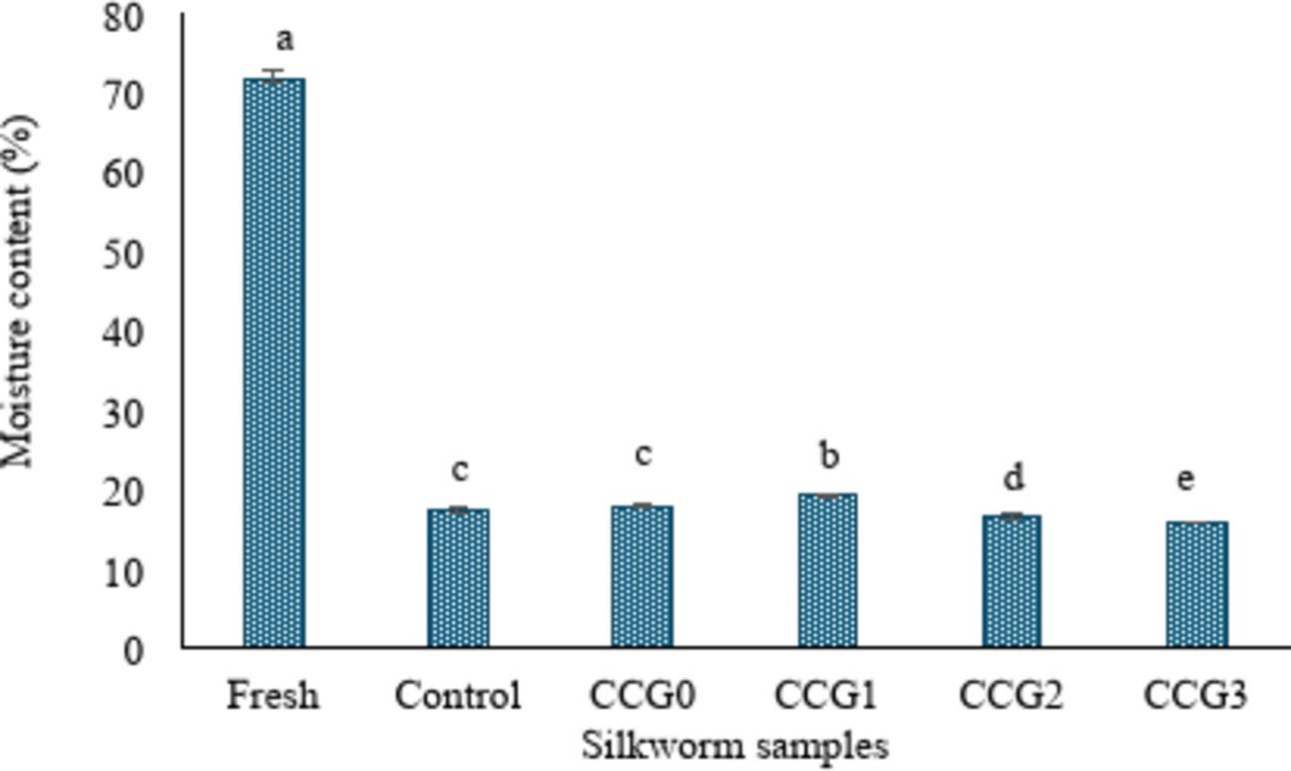
Moisture content of silkworm samples.

The result of moisture content of silkworm was shown on Fig. 5. As a result of the mass loss percentage increased throughout the drying process, the moisture value of the fresh sample was highest (71.5%) compared to dried samples. The moisture of dried samples gradually decreased from the control to CCG3. Specifically, the moisture of dried silkworms fluctuated from 19.28% in CCG1 (the highest moisture content of dried-samples) and 15.58% in CCG3 (the lowest moisture content among dried-samples) (*P* < 0.05). This result indicated the effect of coatings on silkworm during drying process, specifically, all samples were treated with the same drying condition such as temperature, time, and airflow forced. CCG3 indicated the lowest moisture content compared to the other coatings. This result might reveal that the coating formular showed good characteristics to prevent shrinkage and retain the original cell structure during moisture loss in drying process. This result also suggests that the coating could save more energy and time to reach optimized moisture in the drying process. Ando et al.^44^compared two different drying methods of freeze-drying and hot air-drying on Silkworm Larvae (*Bombyx mori*). The author reported that freeze-drying can keep the original silkworm appearance while significant shrinkage structure occurred in hot air-drying. Therefore, the application of coating dried products opens a new direction for agriculture sustainability.

#### Metabolism profile of dried silkworm

In this study, the primary metabolic process of silkworms was identified according to the metabolomic database profiles. 109 ingredients were detected (Table S3), mainly organic acids, fatty acids, esters, sugars, nitrogenous compounds, and other important metabolites. Silkworms were coated with different solutions, resulting in differing relative percentages of each component. The sugar and sugar alcohol group included 34 components, with monosaccharides being the most prevalent type of main metabolite. Among these components, trehalose-8TMS, maltose-meto-8TMS, arabitol-5TMS, xylitol-5TMS, glucose-meto-5TMS, and sorbitol-6TMS occupied higher relative percentages in all silkworm samples. Besides, a total of 18 organic acids, including phosphoric acid-3TMS, fumaric acid-2TMS, malic acid-3TMS, 2-aminopimelic acid-3TMS, oxalic acid-2TMS, and citric acid-4TMS, were detected in all samples at high relative concentrations. In general, fresh silkworms had a lower relative concentration of organic acids compared to dried silkworms (*P* < 0.05). Key primary metabolites identified included fatty acids/esters like stearic acid-TMS, kynurenic acid-TMS, and palmitic acid-TMS. Silkworms were characterized by high sources of protein, fat, minerals and vitamins with 21.5% of protein sources, 13.0% of fat, and 6.70% of carbohydrate per 100% edible part^45^. The released results revealed that there were significant differences between two groups of CCG2, CCG3 and the others. Specifically, CCG2 and CCG3 represented higher relative concentrations (*P* < 0.05) during metabolite process in comparison to other groups with components such as Phosphoric acid-3TMS, Malic acid-3TMS, 2-Hydroxyglutaric acid-3TMS, Glucuronic acid-meto-5TMS, Xylitol-5TMS, Allose-meto-5TMS, Galactose-meto-5TMS, Glycerol-3TMS, Glutamine-3TMS, Lysine-4TMS, Uracil-2TMS, Serine-3TMS, Leucine-2TMS, and Tryptophan-3TMS. In detail, CCG2 was more dominant in amino groups (Leucine-2TMS, Tryptophan-3TMS) while CCG3 was more dominant in carbohydrates and nitrogenous (Malic acid-3TMS, Galactose-meto-5TMS, 2-Hydroxyglutaric acid-3TMS). Additionally, the analysis indicated that both the quantity and relative percentage of substances, as well as the qualitative, were lower in fresh samples compared to dried samples (*P* < 0.05). Some components, such as succinic acid-2TMS and glutamine-3TMS, showed higher concentrations in coated silkworms than in uncoated ones (*P* < 0.05). These differences showed the influences of thin film layer on the surface of silkworm during drying process, which were shown by increasing the relative concentration of main components. The drying process was confirmed by the major change in sensory properties^46^ or texture, volatiles, and appearance of dried products^47^. The most significant factor leads to these changes was from Maillard response. This reaction also resulted in metabolism changes which was confirmed by the relative percentage alternatives of groups such as fresh and dried silkworms (CCG0, CCG1, CCG2, CCG3) in this study. The result suggests the application of coating for drying process had a significant effect to prevent the nutrient degradation of dried products.

**Fig. 6:**
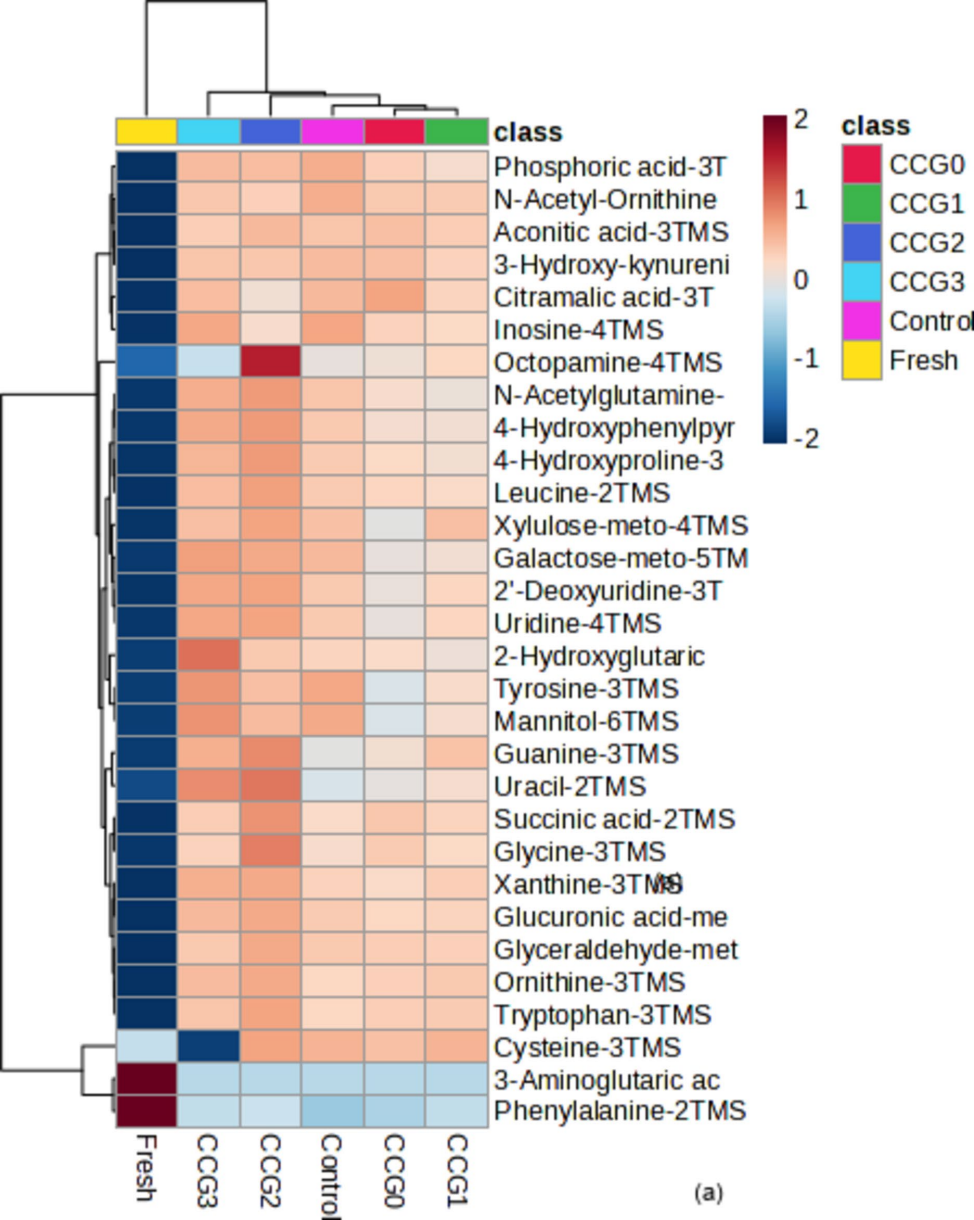

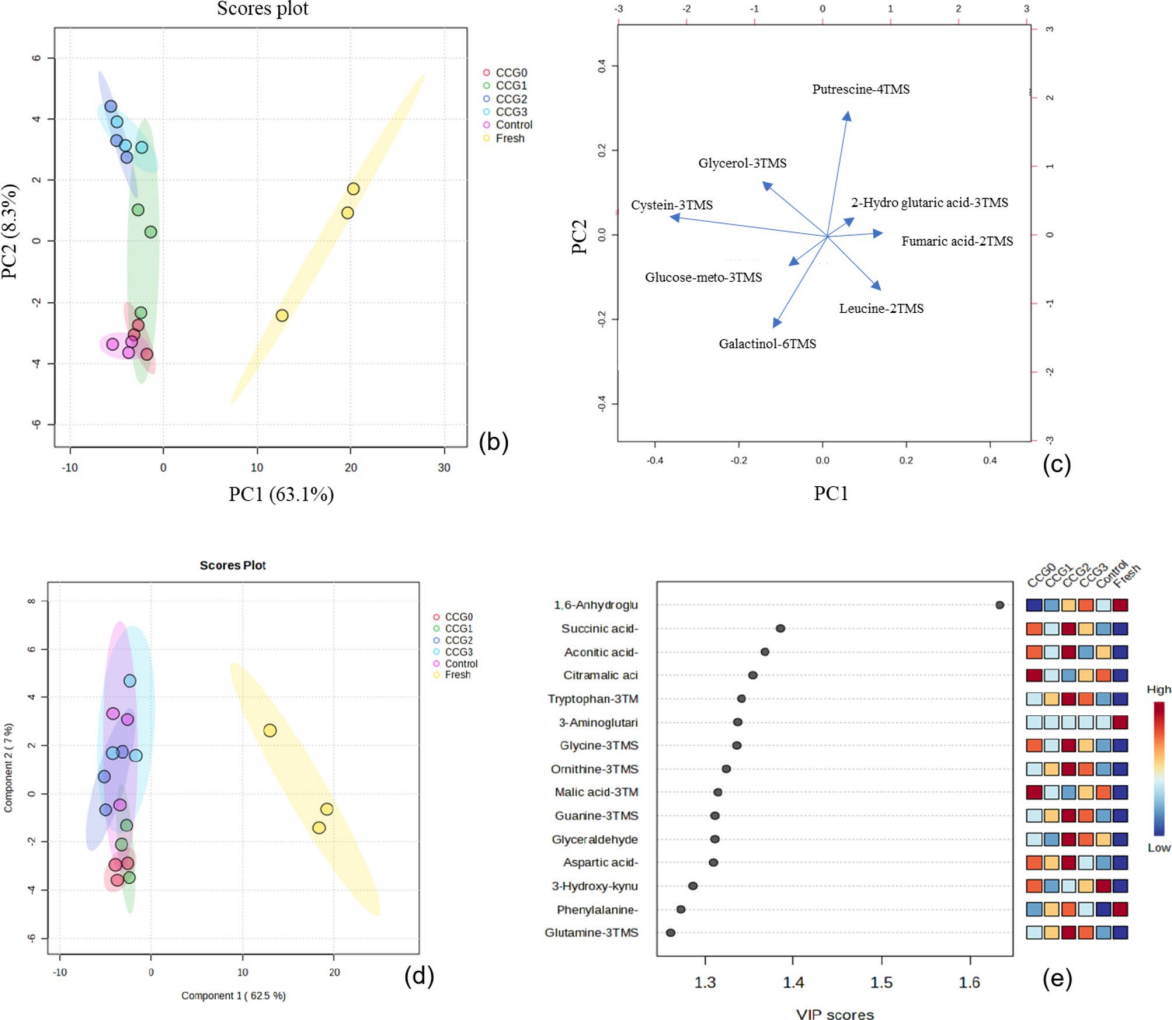
Heatmap (**a**), PCA score plot (**b**), biplot (**c**), PLS-DA score plot (**d**) and important features (**e**) of uncoated and coated silkworms.

The metabolic profile heatmap (Fig. 6a) shows that the silkworm samples were divided into six groups: group A (fresh), group B (CCG3), group C (CCG2), group D (Control), group E (CCG0), and group F (CCG1). The heatmap colors represent the normalized peak intensities of the spectrum, where dark red signifies high intensity, and dark blue represents low intensity. It showed that the peak intensities for most metabolites were greater in the CCG2 and CCG3 samples compared to the other samples.

PCA, an unsupervised statistical method for dimensionality reduction, was performed to identify and thoroughly assess the distinct metabolites, highlighting the overall trends in metabolite changes between uncoated and coated dried silkworms. The PCA score plot (Fig. 6b) illustrated how different treatments affected the metabolic components of silkworms. Notably, the CCG1, CCG2, and CCG3 samples showed a more significant impact compared to the other treatments. The CCG1 sample was mainly aligned with PC2 (8.3%), while the fresh sample predominantly aligned with PC1 (63.1%), clearly distinguishing silkworms before and after being dried. The PCA biplot (Fig. 6c) offered further insight into the metabolic profiles of silkworms. Specifically, the metabolic profile of the CCG2 group was characterized by the presence of cysteine-3TMS, while the control group was linked to galactiol-4TMS. In contrast, the CCG1 group featured unique metabolites, including putrescine-4TMS and glycerol-3TMS. These results indicated the significant effect of coatings on the drying process due to their ability to cover the surface of silkworms, which was confirmed to be influenced by direct exposure with heat or airflow in the dryer^44^. The reasons for the significant distinguishing might be from the Maillard reaction leading to the chemical component changes such as oxidation, degradation, or water loss^48^. This metabolism modified the ratio composition on dried products, that were affected by different coatings. These results were confirmed by the change in the moisture content, porosity ratio in this study.

Partial least squares-discriminant analysis (PLS-DA) was employed to assess the metabolic distinctions among silkworms which were shown in Fig. 6d and e. The PLS-DA model showed clear data segregation and robust predictive capability of fresh and dried samples. Figure 6d shows a 2D score plot, revealing distinct data separation and strong predictive performance between fresh and dried samples. Specifically, fresh silkworm was detected along component 1 at 62.5% while dried samples of control and CCG3 were illustrated along component 2 at 7%. The data presented in Fig. 6e identified fifteen key metabolite components that played a significant role in this separation such as glutamine-3TMS, glutamine-3TMS, aspartic acid, glyceraldehyde acid, guanine-3TMS, ornithine-3TMS, glycine-3TMS, tryptophan-3TMS, aconitic acid, and succinic acid. Among samples, CCG2 showed the highest intensity while the fresh show the lowest metabolic profile compared to others. These results are consistent with PCA conclusion.

#### Color change

The visual appearance of silkworm after coating was shown on Fig. S1a. According to, silkworms were covered by thin film which was clearly seen by eye. The color change results of silkworms (Fig. S1b) showed significant differences in the silkworm samples before and after drying compared to the original sample. Overall, all values of L*, a* and b* decrease after drying process. Specifically, the a* and b* values ​​of coated silkworms decreased significantly in comparison with these values of the control sample, as shown by the significant differences among the results obtained, especially CCG2 and CCG3 (*P* < 0.05), meanwhile, there was no difference in L* values.

The *ΔE* values revealed a significant difference between before and after drying process (*P* < 0.05) as it was calculated from L*, a* and b* values. In particular, CCG1, CCG2, and CCG3, the a* and b* values ​​were significantly lower than those of Control and CCG0, meanwhile *ΔE* values were significantly increase. These results suggested that coating components played an important role in the color change of silkworm before and after drying. Further, this result also demonstrated the effect of coating on drying process. The reason for this change might from the Maillard reactions, leading to the formation of melanoid, brown color nitrogenous polymers, and co-polymers, or pigment degradation, which are responsible for the changing of the redness and brightness of dried silkworm^49^.

#### Instance segmentation application on X-ray CT image analysis

The accuracy of image segmentation process.

**Fig. 7:**
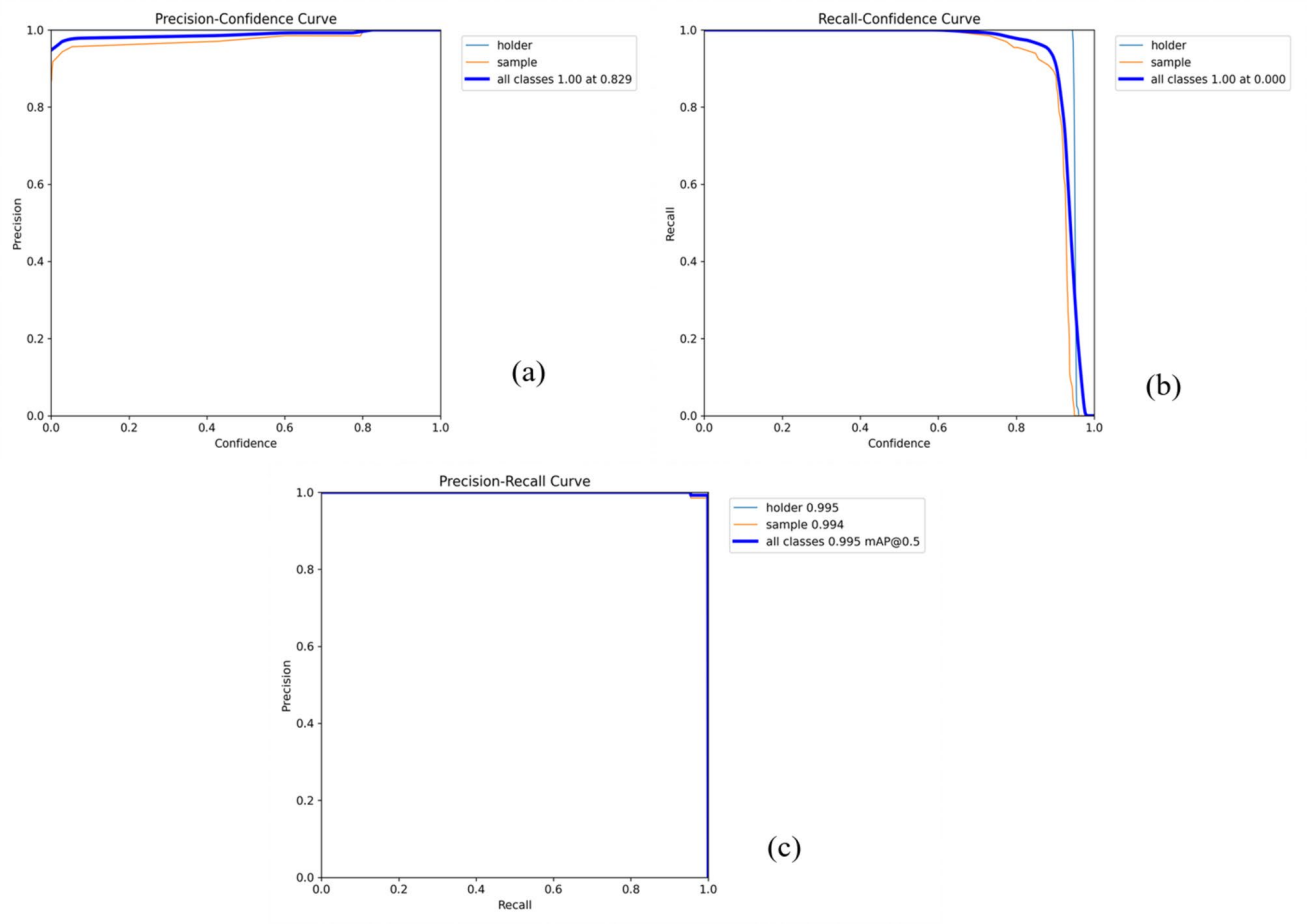
Precision confidence (**a**), recall confidence (**b**), and precision-recall (**c**) curves of X-ray silkworm images.

From previous studies, YOLOv8 represented advantage results in both single and multiple objective classification^50^. In this study, two objectives include the holder and sample which were segmented. As a result, it was shown in Fig. 7, all classes reached a precision of 1.00 at confidence threshold of 0.829 (Fig. 7a) while the recall achieved 1.00 at the lowest confidence threshold (Fig. 7b). In addition, the result about precision-recall curve (Fig. 7c) revealed that mAP values (a mean average precision) reached 0.995 over all classed objects at IoU value (an intersection over union) of 0.5, which were detailed for sample holder at 0.995 and sample 0.994. These results demonstrated a high accuracy in image segmentation of YOLOv8. This finding was similar to the result of Sapkota et al.^50^, which was confirmed by several segmentation methods in both single and multiple segmentation. This YOLOv8 also demonstrated a higher accuracy performance in the condition of low light and time sensitive tasks, which plays an important role in agricultural product quality by machine learning.

The instance segmentation analysis of silkworm samples.

**Fig. 8:**
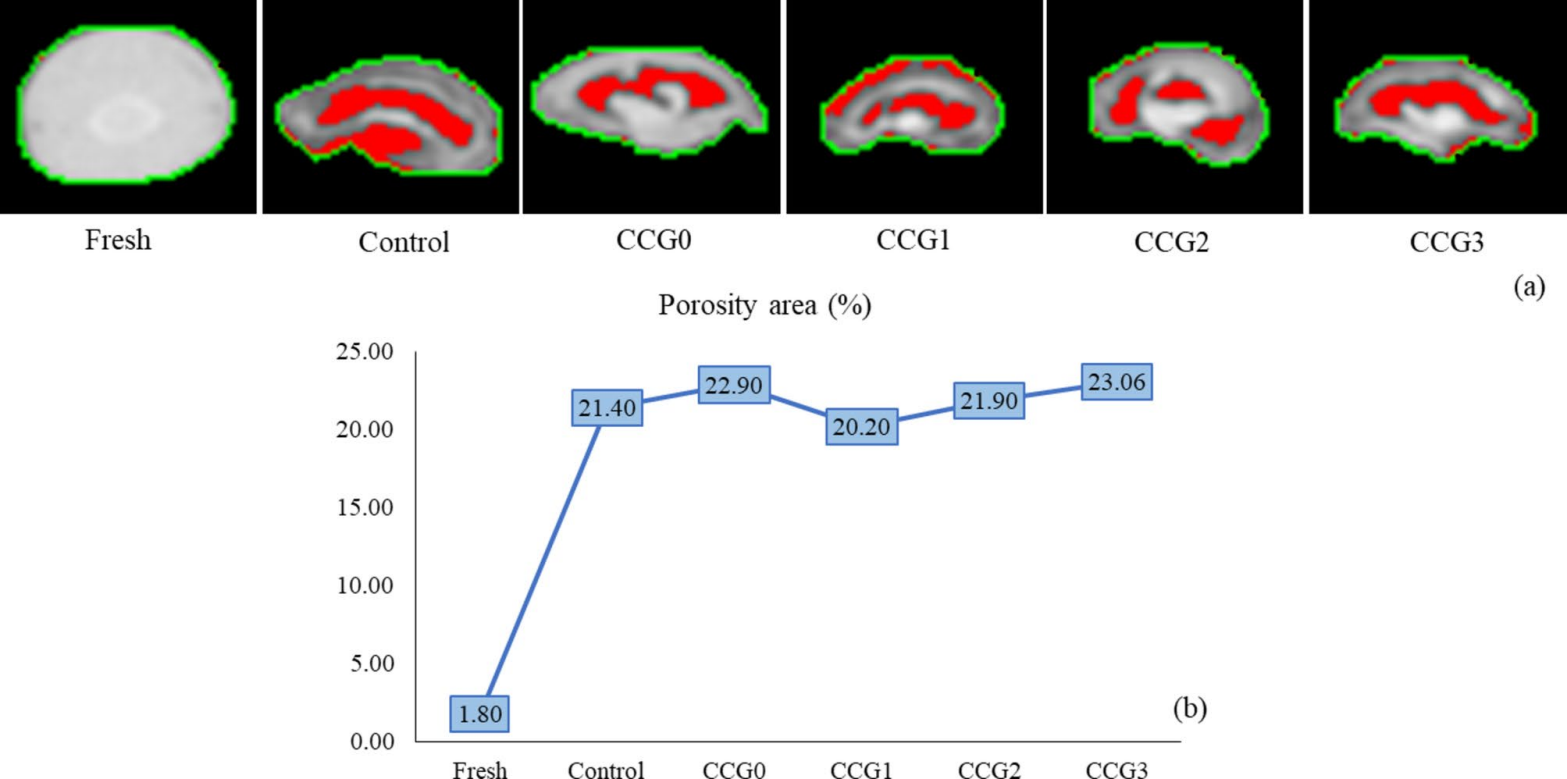
The porosity area of silkworms (as red color), boundary of segmented sample (as green color), images after segmentation and image processing (**a**), and data analysis of porosity area (**b**).

The structural changes of the dried product depend on the product itself, the drying method, and the drying conditions such as temperature, humidity, airflow speed, and drying time. The application of coating film on silkworm surface was first reported in this study. The evaluation of the microstructural changes in fresh and dried silkworms demonstrated the influence of the coating film on the silkworm quality and the efficiency of the drying process. The microstructure of the cell changes based on the temperature difference between the drying airflow and the surface or core temperature of the cell tissue, as a result, the porosity of the cells also varies^51^. In this study, all silkworm samples were dried under the same conditions. After that, X-ray images were evaluated using image processing. The results of X-ray CT in this study indicated the different sizes of the red color which represented for the porosity ratio in images (Fig. 8a) and average porosity ratios (Fig. 8b) among silkworm samples. As can be seen from the Figs., the porosity ratio of the fresh sample was very low and almost unobservable (1.80%). In contrast, the porosity area of dried samples was 10 times higher, with the CCG3 and CCG0 samples being the largest at 23.06% and 22.90%, respectively, while the control and CCG1 samples were smallest at 21.40% and 20.20%. These results suggest that using edible coatings for fresh samples might reduce the time of drying process and save energy. Ando et al. explained the difference in temperature of the drying airflow and the surface of tissue leads to altering the cell shrinkage during drying process, and stiffness in the cell tissue quickly^44^. This limits the water evaporation of internal moisture and hinders the dehumidification of the sample during drying process through the cell^44^. Based on this hypothesis, the mechanism of edible coating might be that the covering of a thin film on the sample surface, leading to reduce the temperature difference between the hot airflow stream and the cell surface, preventing the stiffness of the cell, and promoting dehumidification adequately.

## Conclusion

In this study, the effect of the combination of CMC/NCF/N-Ch and GPO on silkworm drying process was determined. The antibacterial activities of these edible coating were also clarified. The results showed that the various concentrations of GPO affected the physicochemical characteristics of the coating such as pH, TSS, and viscosity values, especially force curve and elastic modulus values which represent the elastic on the surface of the film. In addition, the result of thermal analysis reveals reduced weight loss with higher GPO concentrations, particularly in the CCG3 film. The CCG3 film exhibited the strongest antibacterial effects against *Staphylococcus aureus* while effectively maintaining lower moisture content than others. Additionally, CCG3 displayed more extensive metabolic conversion of compounds and showed greater porosity values in dried silkworm. These findings suggest that edible films on dried products offer significant potential, advancing product quality, energy efficiency, and sustainable practices. However, besides positives, the study remained some drawbacks on the film characteristic such as inhomogeneous surface structure, or low antibacterial effects which require high concentration of GPO. Therefore, studies on other essential oils combined with these bases should be deployed in the future.

## Material and methodology

### Chemicals

CMC and NCF were industrial standard and sourced from Wako Pure Chemical Industries and Nippon Paper Industry (Japan). N-Ch with purity 99.9%, average particle size was 80–100 nm, molecular weight at 161 (g/mol) was obtained from Nano Research Element (Haryana, Indian). Glycerol was guaranteed standard, ribitol was special standard, while pyridine, methanol, and chloroform were reagent standard. N-hexane was Wako first grade, and calcium chloride was reagent grade. These chemicals were provided from FUJIFILM Wako Pure Chemical Co., Ltd. (Japan). N-methyl-N-trimethylsilyltrifluoroacetamide (MSTFA), methoxyamine-HCl, and n-alkanes (the qualitative retention time index, 100–200 µg/mL in hexane) were procured through GL Sciences, Co., (Japan), and Restek Corp., (USA), respectively. PDA (potato dextrose agar) and PDB (potato dextrose broth) were obtained through Nissui Pharmaceutical Co., Ltd. (Japan). To ensure high purity for experimental procedures, Millipore water was processed through an Autopure WT101 UV system provided by Yamato Corp., Japan. Additionally, distilled water used in the study was obtained utilizing the automatic water distillation apparatus of Aquarius Advantech GS-200 manufactured by Toyo Seisakusho Kaisha, Ltd., (Japan). These materials and apparatuses were carefully selected to maintain the integrity and reliability of the experimental outcomes.

### Preparation of coatings and films

The stock mixture was developed as follows: 1.0% (*w/v*) of CMC, 0.5% (w/v) of NCF and 0.1%, (*w/v*) of N-Ch powders. These materials were slowly poured into stirred distilled water by water bath (EWS-100RD, AS ONE, Japan) at 850 rpm, 85 ^o^C in 1 h. Then, 0.35% (*v/v*) of glycerol was added to the above solution during the stirring step. After the stirring step finished, the coating solution was kept in the room temperature for cooling down, then, the GPO was added in ratio 0.01; 0.1; 1.0% (*v/v*). Subsequently, the homogeny step by T-25, IKA, (Japan) was used to get final solutions in 10 min and 20,000 rpm. The targeted solutions named CCG0 (CMC, N-Ch/ NCF), CCG1 (CMC, N-Ch/ NCF, GPO 0.01%), CCG2 (CMC, N-Ch/ NCF, GPO 0.1%), and CCG3 (CMC, N-Ch/ NCF, GPO 1.0%). A vacuum Yamato ADP300 (Japan) was used to de-bubbled and ensure uniformity of solutions. To make films, the solution was poured onto a square silicon plate with 20 mL of volume, dried for 8 h at 40 °C in an oven EYELA WFO-520 W (Japan). After drying process, the dried film was removed from the plate and kept in the plastic desiccator at room temperature and 53% RH with calcium chloride to reach constant state for subsequent determinations.

### Silkworm preparation

A total of 125 silkworms and the 3.5 kg wet powder of mulberry were obtained from Ehine Sansyu Co. Ltd., (Japan). The wet powder scroll of mulberry was used to feed the silkworms in 15 days. After the silkworms transfer to the cocoon, the silkworm pupa was collected, then they were divided into five groups as follows: control, CCG0, CCG1, CCG2, CCG3. In this step, these silkworm pupas were treated with distilled water and four kinds of coatings, respectively. After that, they were dried in an incubator (Isuzu Seisakusho Co., Ltd, Japan) at 70 ^o^C, 20% RH, and flow rate at 2.7 m/s in 5.5 h. For metabolism analysis, the dried silkworm pupa was grilled by a grinder (Philips HL-3258, Japan) in 1 min, and then the silkworm powder was kept in a freezer (NF-300 HC, Nihon Freezer, Japan) at -60 ^o^C to prevent of degradation of quality until further experiments.

### Analytical methods

#### Characteristics of the emulsions and films

##### pH, viscosity of emulsions

A LAQUA twin pH meter (Horiba, Japan) and a viscometer (Viscometer TV200, Toki Sangyo Co., Ltd, Japan) were used to measure the pH and viscosity of the emulsions. Basically, 1 mL for pH and 1.1 mL for viscosity of coatings was used to measure the value at room temperature, three replications.

##### Color, thickness of films

The color of the film was characterized at various angles by a CR-20 color reader (Konica Minolta Inc., Japan), with five values recorded for each film sample. The measurements were based on the Commission Internationale de l’Eclairage (CIE) color scale, where the L*, a*, b*, and Δ*E* color systems included. Specifically, L* values are from 0 to 100, representing the spectrum from diffuse from black to white, with white values potentially exceeding 100. The a* values describe the color between red and green, while the b* values indicate the color between yellow and blue. The total color change (∆*E*) was determined using Eq. (1), described by Van, et al.^15^. This comprehensive approach allows for precise quantification of color changes in the film, facilitating a better understanding of its optical properties.

1 $$\:\varDelta\:E\:=\:\sqrt{{({L}_{\text{o}}-L\text{*})}^{2}+{\left({a}_{\text{o}}-a\text{*}\right)}^{2}+\:({{b}_{\text{o}}-b\text{*})}^{2}}$$

The values of *L*_o_, *a*_o_, and *b*_o_ were for the white plate, meanwhile L*, a*, and b* were for the membranes, five replications.

As for thickness, a digital micrometer (Series 102–707, Mitutoyo Corporation, Japan) was eliminated to record the film thickness (µm), using to the accuracy at 0.001 mm and five points randomly per film.

##### Scanning electron microscopy (SEM)

The cross-section and surface of films were clarified by SEM system (SU3500, Hitachi High-Tech, Corp., Japan). Basically, a small portion of the film was put into nitrogen liquid for freezing, then, the films were broken out to several pieces. Next steps, the broken films were marked to record the broken points. For imaging, the fractured films were mounted on conductive double-sided tape and coated with a fine gold layer using a plasma coater (HPC-1SW, Vacuum Device Co., Japan). Images were captured at 15.0 kV voltage, with a magnification of 300x and a width of 100 μm for the surface view, and at 15.0 kV, 1.00 k magnification, and 50.0 μm width for the cross-sectional view.

##### Atomic force microscopy (AFM)

Film roughness was measured by AFM system (Hitachi 5200 S, Japan) conducted in tapping mode of Nano Navi Application. Coatings were similarly formulated by adding them (CCG0, CCG1, CCG2, and CCG3) to the fresh carbon tape before imaging. The cantilever of SI-DF3P2 was chosen with a scanning frequency around 1 Hz, and an area scanning of 5 μm × 5 μm. There were two types of measurements including roughness surface and force mapping. Before the measurement, the performance of Q-curve in DFM mode was evaluated and performed the approach to get the standard curve. After that, AFM and the force mapping mode were set up to measure the force curve and elastic modulus of film samples. The time for analyzing one sample was 5 h, which depended on the scan area of sample setting. The force curve values indicated the indentation force acting on the sample at a given penetration depth which can represent the stiffness and adhesion properties of the sample, characterized by *F* values (nN). The modulus of elasticity (*E*_*s*_, MPa) was calculated by converting the measured force curve into a load-indentation curve and fitting a predetermined DMT contact model to the load indentation curve. Measurements were taken from 10 distinct areas, and the values were calculated using Eq. (2) to (3) as detailed below.

2 $$\:F\:=\:\alpha\:\:.\:{\delta\:}^{\frac{3}{2}}\:+{F}_{ad}$$

3 $$\:{E}_{s}\:=\frac{1\:-\:{V}_{s}^{2}}{\frac{4\sqrt{R}}{3.\alpha\:}\:-\:\frac{1\:-\:{V}_{t}^{2}}{{E}_{t}}}$$

*F*: Force load; *α*: Coefficient; *V*_*s*_: Sample poisson’s ratio; *δ*: Penetration depth; *F*_*ad*_: Adhesion force; *E*_*s*_: Sample elastic modulus; *R*: Probe curvature radius; *E*_*t*_: Probe elastic modulus; *V*_*t*_: Probe poisson’s ratio.

##### Thermal analysis

The thermogravimetry (TG) and differential thermogravimetric (DTG) curve were measured with a thermogravimetric analyzer (TGAQ500, TA Analyzer, USA) under a nitrogen (N_2_) atmosphere. The sample mass was about 5 mg, and the test temperature range was from 50 ^o^C to 450 ^o^C.

#### Determination of antimicrobial activity

The bacterium *Staphylococcus aureus* was selected for testing. *Staphylococcus aureus* was shown to alive (but may not grow) at low-moisture foods and environments for extended periods of time^52^. Besides, *Staphylococcus aureus* is the most well studied in silkworm models^53^. Firstly, the bacteria were cultured in a sterile environment using tryptic soy broth (TSB) at the recommended concentration of manufacturer for 24 h at 37 °C. Subsequently, 10 µL of the bacterial solution at a concentration of 5 × 10^5^ (spores/µL) was used to test the antibacterial resistance of the coating solutions. To prepare for the experiment, 10 mL of sterilized tryptic soy agar (TSA) solution combined with 5 mL of sterilized distilled water (control) or 5 mL of the coating solution, and then 10 µL of the bacterial solution, were placed in the same test tube. The solution in the test tube was stirred at 1200 rpm for 10 s for homogenization. Then, the solution in the test tube was transferred onto plastic petri dishes, incubated at 37 °C, and the count of colony-forming units was examined after 24 h. The colony count was automatically determined using colony counter software (Shashin Kagaku PSF-1000, Japan).

### Silkworm pupae application

#### Moisture content

The moisture content of samples was determined by drying samples at 105^o^C in 24 h. The weight in initial and final day was recorded, then moisture content was calculated by Eq. (4):

4 $${M_{\text{C}}}={\text{ }}({M_0}\, - \,{M_{\text{1}}}) \times {\text{1}}00/{M_0}$$

where *M*_C_ is the moisture content (%), *M*_0_ is the weight before drying (g), and *M*_1_ is the weight of final drying time (g).

#### Color change

The color changes of silkworm were measured using CR-20 (Konica Minolta Japan Co., Ltd.) by evaluating *∆E*, L*, a*, and b* values. The *∆E* value was determined using Eq. (1), where *L*_o_, *a*_o_, and *b*_o_ represent the values before coating while L*, a*, and b* correspond to the values after coating and drying, five replications.

#### Metabolism profile of dried silkworm

The metabolism process of silkworms was reflexed using a GC-MS/MS system (Shimadzu, Kyoto, Japan). The gas chromatography (Nexis GC 2030, Shimadzu Corporation) was outfitted with a DB-5 column (Shimadzu Corp., USA), with specifications of a 0.25 mm inner diameter, a 30 m length, and a film thickness of 1.0 μm. A liquid injection system delivering 50 µL/s was employed, with the injection port temperature set at 250 °C. The column temperature program began with a 4 min hold at 100 °C, then rose to 320 °C over 11 min in spitless mode. Helium served as the carrier gas, moving at a linear velocity of 39.0 cm/s and a pressure of 83.7 kPa. Mass spectrometry was performed across a range of 45 to 600 m/z, with the ion source and interface temperatures at 200 °C and 280 °C, respectively. The temperature of injection port was maintained at 280 °C. All the setting was followed by Van et al.^25^.

In the initial analysis phase of analysis, a smart metabolite database file from Shimadzu Corporation (Tokyo, Japan) was employed to calculate retention times using 100 µg/mL of n-alkanes. After calibration, the generated data file was integrated with the sample scanning method file from the smart metabolite database to create a customized scanning protocol for sample analysis. The original method targeted 467 specific compounds. Subsequent steps involved generating acquisition and batch files to measure the samples, which included both blank and silkworm samples. Blank vials were analyzed first to verify no contamination, followed by sample measurements, with each sample tested in triplicate. Relative proportions of the tentatively identified compounds were determined by the height ratio of ribitol, (the internal standard) to the detected components. Silkworm powders were prepared by grinding samples with a grinder for further analysis.

For sample preparation, fresh silkworms and dried silkworms were grinded by hand grinder prior to scanning. Then, 10 mg of grinded silkworms were initially diluted with 10 µL of ribitol solution (1 mg/mL). Then, 1 mL of Millipore water and a methanol-chloroform mixture (1:2.5:1 *V/V/V*) were added. Samples were ground using Micro Smash MS-100 R grinder (Tomy Seiko, Japan) at 4600 rpm for 5 min, then centrifugation by Kubota 5922 (Yama P Co. Ltd, Japan) at 15,000 rpm for 3 min at 4 °C. Subsequently, 900 µL of the sample and 400 µL of Millipore water were added, stirred, and centrifuged again. Next, the supernatant (200 µL) was extracted, heated, and stirred to evaporate methanol using SPD1030A-115 equipment (Thermo Fisher Scientific, USA), and a speed vacuum concentrator. In derivatization step, methoxyamine-HCl (100 µL) in pyridine (20 mg/mL) was incorporated into the vacuumed samples. The mixture was heated, and shaken at 30 °C and 1200 rpm for 90 min. Afterward, MSTFA solution (50 µL) was introduced, and the mixture was further shaken at 37 °C and 1200 rpm for 30 min. After a final centrifugation at 20 °C and 15,000 rpm for 3 min, 100 µL of the solution was transferred to a glass vial with a lid (Shimadzu Corp., Japan), and the vials were arranged in sequence on the tray holder. The profiles of the compounds present in the silkworms were instantly analyzed using the smart metabolite database. Each sample was processed in triplicate.

#### Analyzing X-ray CT images by using instance segmentation and image processing

X-ray CT imaging plays a crucial role in non-destructive, non-invasive 3D imaging which allows researchers to visualize and quantify internal modifications in samples, such as changes in tissue density, cell wall integrity, and porosity, without damaging the samples. With this purpose, X-ray CT was invaluable function for studying the microstructural changes of biomaterials over time^54,55^. In this study, the drying process had directly affected internal structure of silkworm which was caused by the water loss and microstructure change such as porosity rate. Specifically, the porosity indicates the void space or the pore space volume as a fraction of the total sample volume^56^. Moreover, the change of internal structure was represented by ratio of porosity inner samples before and after being dried. After taking the X-ray CT images, images were analyzed by using AI and image processing which was confirmed by high accuracy and faster than manual. The workflow was shown on Fig. S2.

##### Image collection

The purpose of this method is to determine the differences between the white and black pixel ratio in the silkworm samples, which represented the solid and empty parts in cell tissue structure after the drying process. The process of X-ray CT imaging of fresh and dried silkworms was performed using a CosmoScan FX scanner (Rigaku Corporation, Tokyo, Japan). The operational parameters were configured with a voltage of 90 kV, a current of 88 µA, a 0.06-mm Cu filter, a 0.5-mm Al filter, and a field of view (FOV) of 72 mm for standard CT scanning. The samples were positioned in holding tubes, and the scanning duration was set at 2 min with a pixel size of 144 μm. A total of 2500 images for all samples with resolutions of 256 × 256 were collected and analyzed. There were five samples including control, CCG0, CCG1, CCG2 and CCG3. These images were taken automatically using X-ray CT system.

##### Image annotation

In this study, there were two objectives which were determined including the holder and the sample. After annotation (a total of 557 images), the images were augmented and splitting in the dataset. To facilitate model training and validation, images were divided randomly into training and validation with 486 images for training and 71 images for validation, respectively.

##### Model training

In this study, the YOLOv8s model was selected for training AI model. The model was trained via transfer learning^57^ by fine-tuning pre-trained weights learned on the COCO dataset^16^. Firstly, the AI model was trained using the PyTorch 2.1.0 deep learning framework on an Ubuntu 20.04 operating system. The training process involved utilizing CUDA version 11.8 with the Intel(R) Core(TM) i7-11800 H, 2.30 GHz serving as the central processing unit (CPU), and the NVIDIA 3070 was handling the graphics processing unit (GPU) tasks for image training. In addition to the training process, the YOLOv8s model, with 11.2 million parameters, was configured with an initial learning rate of 0.01, whereas the momentum and weight decay used were 0.937 and 0.0005 respectively. The training batch size was set to 8 images, and the total number of iterations was fixed at 150. These parameter settings were chosen to optimize the speed of the training process while minimizing the chances of overfitting the model to the training dataset.

Model evaluation: the calculation was performed by Eq. (5) (6) (7) (8) as following Sapkota et al.^50^

5 $$\:\text{P}\text{r}\text{e}\text{c}\text{i}\text{s}\text{i}\text{o}\text{n}=\:\frac{TP}{TP+FP\:}$$

6 $$\:\text{R}\text{e}\text{c}\text{a}\text{l}\text{l}=\:\frac{TP}{TP\:+FN}$$

7 $$\:\text{I}\text{o}\text{U}=\:\frac{Area\:Overlap}{Area\:Union}\:=\:\frac{TP}{FP\:+TP\:+FN}$$

8 $$\:\text{m}\text{A}\text{P}=\left(\frac{1}{K}\right)\sum\:_{i=0}^{k}\left(AP\right)n$$

In the calculation, *TP*, *FP*, and *FN* deputize true positive, false positive, and false negative object instances respectively. In addition, *k* deputizes the total number of objects while (*AP*)*n* represents the average precision calculation for n objects with *AP* refers to the area of the precision-recall curve for a given object.

##### Deployment/testing

After the training model, the resulting model was deployed on the remaining images. The use of image processing, then, was applied to calculate the porosity and solid area on images after segmented images. The porosity area on silkworm samples were calculated following Eq. (9):

9 $$\:Po\:=\frac{The\:porosity\:area\:\:inside\:segmented\:samples}{Total\:area\:segmented\:samples}\text{x}\:100$$

Where *Po* is ratio of the porosity area of segmented images.

### Statistical analysis

R software (version 4.2.2) was used to analyze the results. Treatment effects were assessed through analysis of variance (ANOVA), and significant differences were determined using Tukey’s test (*P* < 0.05). Metabolic profile was analyzed using Metaboanalyst software 5.0. The data used in this study are available in supplementary materials.

## Electronic supplementary material

Below is the link to the electronic supplementary material.

Supplementary Material 1

## Data Availability

Data will be made available on reasonable requests through the corresponding author.
