# Supplementary material for: Application of green pomelo peel essential oil-based carboxymethylcellulose coatings reinforced with nano chitosan and nano cellulose fibers during the drying process on dried silkworms
Source: Sci Rep. 2025 Mar 13;15:8749. doi: 10.1038/s41598-025-93243-7 (PMC11906623; doi:10.1038/s41598-025-93243-7)
Supplement: Supplementary file 1 — Supplementary Material 1 [file 41598_2025_93243_MOESM1_ESM.docx]

**Effect of green pomelo essential oil on co-reinforcing nano chitosan and nano cellulose fiber coatings: An application on dried silkworm**

Tran Thi Van^1^, Fumina Tanaka^2^, Meng Fanze^1^, Mohammad Hamayoon Wardak^1^, Dong Pham Thanh^2^, Ata Aditya Wardana^3^, Laras Putri Wigati^2^, Xirui Yan^2^, Fumihiko Tanaka^2^

^1^Graduate School of Bioresource and Bioenvironmental Sciences, Kyushu University, 744, Motooka, Nishi-ku, Fukuoka-shi, Fukuoka 819-0395, Japan

^2^Faculty of Agriculture, Kyushu University, W5-874, 744, Motooka, Nishi-ku, Fukuoka-shi, Fukuoka 819-0395, Japan

^3^Department of Food Technology, Faculty of Engineering, Bina Nusantara University, Jakarta 11480, Indonesia

**Figures**


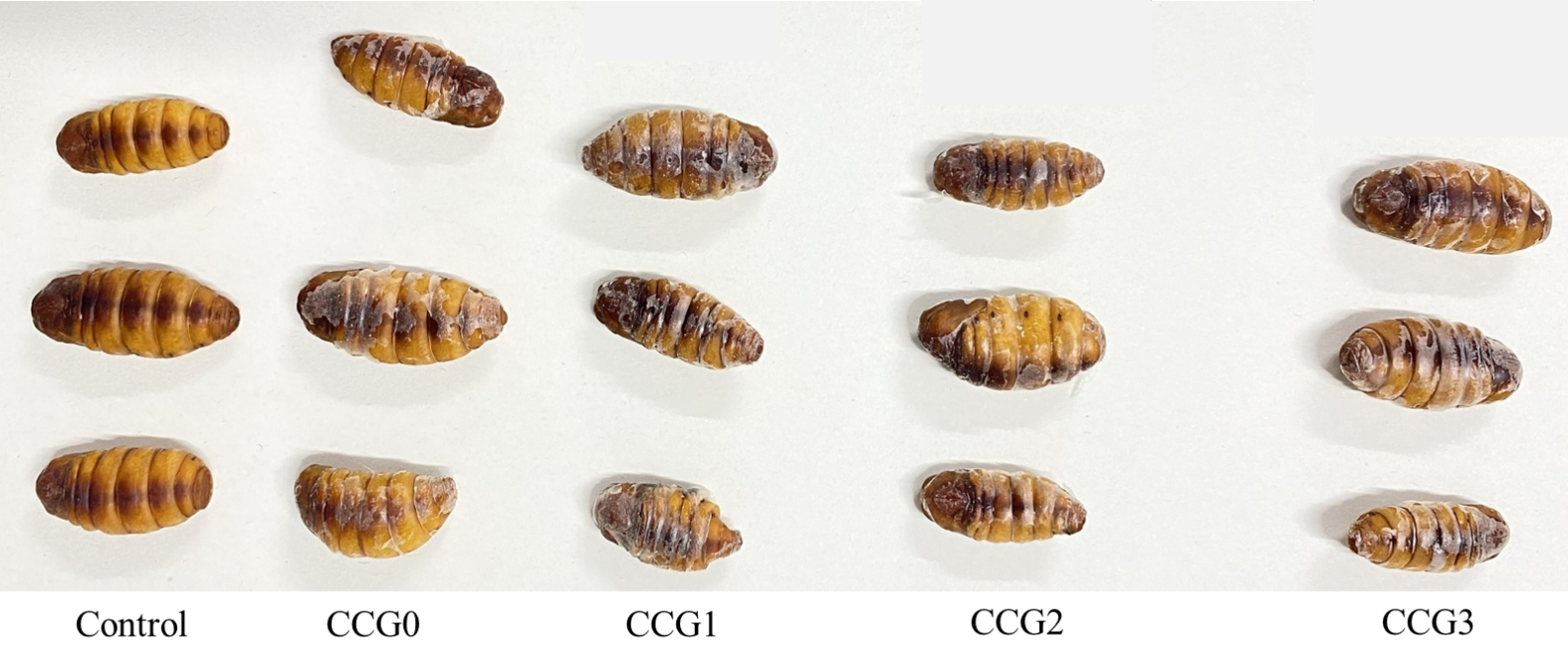


(a)

|  |  |
| --- | --- |
|  | (b) |

Fig. S1 Visual (a) and color change (b) of silkworm samples before and after drying process
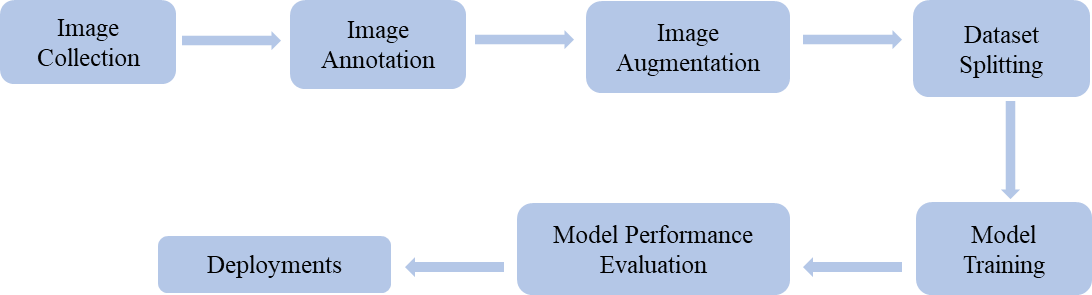


(a)


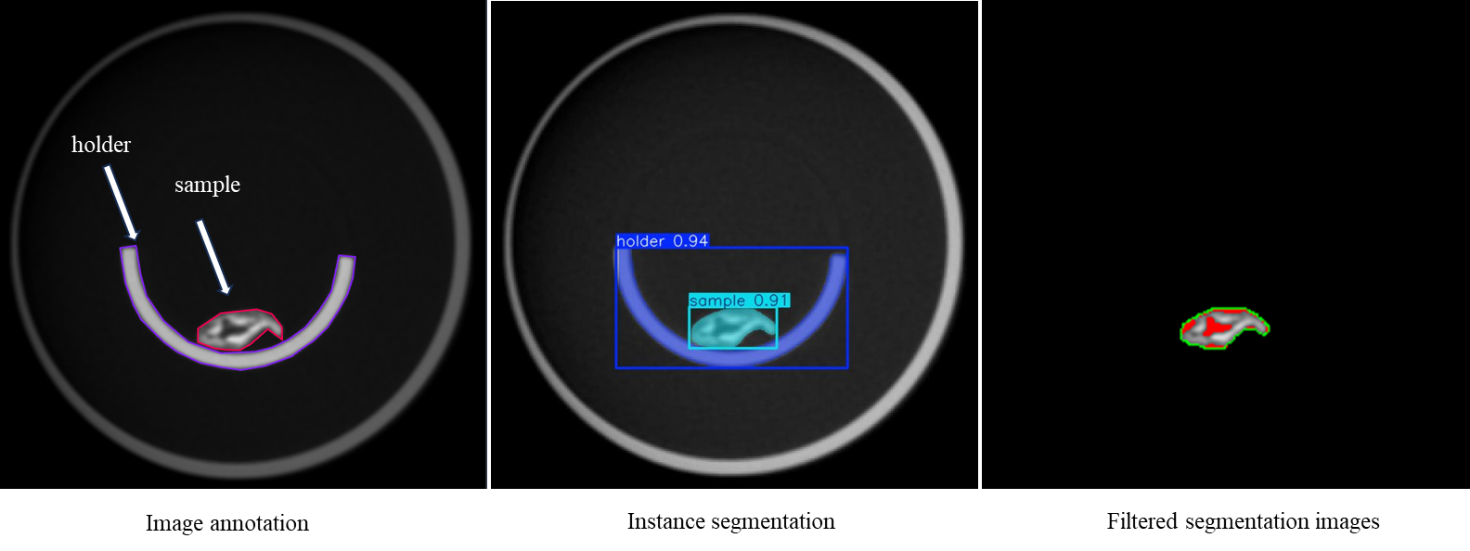


(b)

Fig. S2 Workflow process (a) and steps of annotation, segmentation, and filtered segmentation (b) of silkworm X-ray CT images

**Tables**

Table S1 Coating and film characteristics

|  | **CCG0** | **CCG1** | **CCG2** | **CCG3** |
| --- | --- | --- | --- | --- |
| pH | 9.02^b^±0.04 | 9.30^a^±0.02 | 9.26^a^±0.01 | 9.24^a^±0.02 |
| Viscosity | 141.77^b^±5.76 | 160.77^a^±2.00 | 161.53^a^±0.24 | 167.90^a^±2.63 |
| TSS | 1.67^a^±0.05 | 1.60^b^±0.00 | 1.60^b^±0.00 | 1.60^b^±0.00 |
| Film thickness (mm) | 0.050^a^±0.008 | 0.064^a^±0.003 | 0.056^a^±0.006 | 0.052^a^±0.004 |
| *L** | 98.12^a^±0.10 | 98.28^a^±0.10 | 98.14^a^±0.17 | 98.00^a^±0.40 |
| *a** | 0.32^a^±0.10 | 0.34^a^±0.05 | 0.40^a^±0.06 | 0.40^a^±0.17 |
| *b** | 6.96^a^±0.15 | 7.18^a^±0.12 | 7.20^a^±0.13 | 7.38^a^±0.41 |
| ∆𝐸 | 3.02^a^±0.16 | 3.20^a^±0.10 | 3.26^a^±0.17 | 3.49^a^±0.46 |

Table S2 Temperature and weight loss related to stages of TG curve on cellulose edible films.

| **Films** | **First stage** | | **Second stage** | | **End** | |
| --- | --- | --- | --- | --- | --- | --- |
|  | **Temperature (∘C)** | **Weight loss (%)** | **Temperature (∘C)** | **Weight loss (%)** | **Temperature (∘C)** | **Weight loss (%)** |
| CCG0 | 45–246.4 | 13.65 | 246.4 - 327.5 | 64.01 | 327.5 - 486.0 | 74.6 |
| CCG1 | 45–245.3 | 15.68 | 245.3 - 323.0 | 60.67 | 323.0 - 484.3 | 70.88 |
| CCG2 | 45–242.3 | 15.26 | 242.3-328.3 | 61.34 | 328.3-484.4 | 70.35 |
| CCG3 | 45–249.0 | 11.58 | 249.0-325.6 | 53.05 | 325.6-484.0 | 61.93 |

Table S3 Metabolomic profile on GC-MS/MS of coated and uncoated silkworm

|  | Fresh | Control | CCG0 | CCG1 | CCG2 | CCG3 |
| --- | --- | --- | --- | --- | --- | --- |
| **ORGANIC ACIDS** | | | | | | |
| Oxalic acid-2TMS | 0.047±0.006 | 0.051±0.010 | 0.047±0.007 | 0.045±0.006 | 0.048±0.009 | 0.053±0.009 |
| Phosphoric acid-3TMS | 3.515^c^±0.364 | 8.838^a^±0.565 | 7.950^a^±0.138 | 7.540^b^±0.585 | 8.425^a^±0.210 | 8.466^a^±0.489 |
| Succinic acid-2TMS |  | 0.025^c^±0.001 | 0.029^b^±0.001 | 0.026^b^±0.001 | 0.038^a^±0.001 | 0.028^b^±0.002 |
| Glyceric acid-3TMS | 0.012±0.000 | 0.043±0.002 | 0.040±0.000 | 0.039±0.002 | 0.054±0.002 | 0.045±0.004 |
| Fumaric acid-2TMS | 0.027^c^±0.004 | 0.188^a^±0.032 | 0.195^a^±0.019 | 0.128^b^±0.038 | 0.137^b^±0.025 | 0.185^a^±0.023 |
| 2-Aminopimelic acid-3TMS | 0.564±0.254 | 1.429±0.089 | 1.361±0.071 | 1.246±0.028 | 1.460±0.005 | 1.311±0.042 |
| Citramalic acid-3TMS |  | 0.010^b^±0.001 | 0.012^a^±0.001 | 0.009^c^±0.001 | 0.007^c^±0.000 | 0.010^b^±0.000 |
| Malic acid-3TMS | 0.027^c^±0.006 | 0.078^a^±0.005 | 0.091^a^±0.006 | 0.067^b^±0.003 | 0.059^b^±0.002 | 0.077^a^±0.003 |
| Aspartic acid-3TMS | 0.007±0.000 | 0.017±0.001 | 0.020±0.001 | 0.019±0.001 | 0.023±0.001 | 0.019±0.001 |
| Threonic acid-4TMS | 0.015±0.004 | 0.041±0.002 | 0.034±0.001 | 0.034±0.003 | 0.036±0.001 | 0.033±0.002 |
| Ureidopropionic acid-3TMS | 0.011^b^±0.000 | 0.030^a^±0.002 | 0.031^a^±0.002 | 0.021^a^±0.000 | 0.016^b^±0.000 |  |
| Aconitic acid-3TMS |  | 0.012^b^±0.001 | 0.012^b^±0.000 | 0.012^b^±0.000 | 0.013^a^±0.001 | 0.011^b^±0.001 |
| Isocitric acid-4TMS | 0.013^b^±0.000 | 0.020^a^±0.001 | 0.018^a^±0.001 | 0.016^a^±0.001 | 0.018^a^±0.001 | 0.017^a^±0.001 |
| Citric acid-4TMS | 0.773^b^±0.345 | 1.923^a^±0.119 | 1.825^a^±0.103 | 1.660^a^±0.041 | 1.926^a^±0.008 | 1.717^a^±0.051 |
| Citric acid-d4-4TMS | 0.013^b^±0.006 | 0.032^a^±0.002 | 0.030^a^±0.002 | 0.028^a^±0.000 | 0.033^a^±0.000 | 0.028^a^±0.001 |
| Myristic acid-TMS | 0.019±0.001 | 0.024±0.004 | 0.016±0.001 | 0.015±0.004 | 0.016±0.002 | 0.021±0.004 |
| Pantothenic acid-3TMS | 0.034±0.008 | 0.085±0.005 | 0.075±0.003 | 0.075±0.004 | 0.078±0.003 | 0.074±0.006 |
| **FATTY ACIDS AND ESTERS** | | | | | | |
| Stearic acid-TMS | 0.644±0.099 | 0.699±0.129 | 0.554±0.008 | 0.550±0.096 | 0.559±0.029 | 0.664±0.119 |
| Kynurenic acid-2TMS | 0.066±0.012 | 0.116±0.010 | 0.100±0.001 | 0.090±0.010 | 0.100±0.002 | 0.102±0.008 |
| Palmitic acid-TMS | 1.322±0.199 | 1.476±0.296 | 1.131±0.022 | 1.141±0.193 | 1.232±0.077 | 1.447±0.292 |
| **NITROGENOUS** | | | | | | |
| 2-Hydroxyglutaric acid-3TMS | 0.010^e^±0.000 | 0.384^c^±0.014 | 0.338^d^±0.011 | 0.266^d^±0.029 | 0.444^b^±0.011 | 1.297^a^±0.053 |
| 2-Hydroxybutyric acid-2TMS | 0.037±0.005 | 0.041±0.008 | 0.037±0.005 | 0.036±0.005 | 0.037±0.007 | 0.041±0.007 |
| Dihydroorotic acid-3TMS | 0.187^b^±0.075 | 0.460^a^±0.024 | 0.424^a^±0.022 | 0.371^a^±0.010 | 0.431^a^±0.001 | 0.391^a^±0.015 |
| 4-Hydroxyphenylpyruvic acid-meto-2TMS |  | 0.022^a^±0.001 | 0.017^b^±0.001 | 0.018^b^±0.005 | 0.031^a^±0.004 | 0.028^a^±0.003 |
| **SUGAR ACID** | | | | | | |
| Gluconic acid-6TMS | 0.009±0.000 | 0.010±0.001 | 0.008±0.001 | 0.008±0.001 | 0.014±0.001 | 0.011±0.003 |
| Glucuronic acid-meto-5TMS |  | 0.032^b^±0.003 | 0.029^c^±0.003 | 0.031^b^±0.005 | 0.040^a^±0.006 | 0.036^a^±0.004 |
| **SUGARS AND SUGAR ALCOHOLS** | | | | | | |
| Erythrulose-meto-3TMS | 0.051±0.009 | 0.034±0.019 | 0.007±0.001 | 0.041±0.005 | 0.043±0.000 | 0.026±0.020 |
| Lyxose-meto-4TMS | 0.007^b^±0.006 | 0.034^a^±0.002 | 0.035^a^±0.002 | 0.038^a^±0.004 | 0.044^a^±0.003 | 0.033^a^±0.003 |
| Xylose-meto-4TMS | 0.011^b^±0.003 | 0.022^a^±0.001 | 0.023^a^±0.002 | 0.025^a^±0.003 | 0.028^a^±0.002 | 0.021^a^±0.002 |
| Ribulose-meto-4TMS | 0^b^ | 0.007^a^±0.001 | 0.007^b^±0.000 | 0.008^a^±0.001 | 0.010^a^±0.001 | 0.009^a^±0.000 |
| Ribose-meto-4TMS | 0.011^b^±0.005 | 0.022^a^±0.002 | 0.023^a^±0.001 | 0.025^a^±0.002 | 0.029^a^±0.002 | 0.023^a^±0.002 |
| Xylulose-meto-4TMS | 0^d^ | 0.013^b^±0.001 | 0.009^c^±0.002 | 0.014^b^±0.002 | 0.017^a^±0.002 | 0.014^b^±0.002 |
| Xylitol-5TMS | 0.713^b^±0.006 | 0.718^b^±0.019 | 0.698^b^±0.008 | 0.711^a^±0.012 | 0.737^a^±0.010 | 0.742^a^±0.007 |
| 1,6-Anhydroglucose-3TMS | 0.595^a^±0.003 | 0.584^a^±0.009 | 0.563^b^±0.005 | 0.571^b^±0.006 | 0.587^a^±0.004 | 0.589^a^±0.003 |
| Glycerol 2-phosphate-4TMS |  | 0.006^c^±0.000 | 0.008^b^±0.000 | 0.007^c^±0.000 | 0.009^b^±0.000 | 0.012^a^±0.001 |
| Arabitol-5TMS | 0.692±0.003 | 0.690±0.001 | 0.686±0.003 | 0.689±0.001 | 0.693±0.003 | 0.687±0.003 |
| 2-Deoxy-glucose-meto-4TMS | 0.027^b^±0.01 | 0.061^a^±0.003 | 0.055^a^±0.003 | 0.048^a^±0.000 | 0.054^a^±0.001 | 0.048^a^±0.001 |
| 2-Deoxy-glucose-4TMS | 0.018^b^±0.006 | 0.039^a^±0.002 | 0.036^a^±0.002 | 0.032^a^±0.000 | 0.039^a^±0.000 | 0.033^a^±0.001 |
| Tagatose-meto-5TMS | 0.031^b^±0.002 | 0.093^a^±0.005 | 0.080^a^±0.002 | 0.076^a^±0.021 | 0.082^a^±0.004 | 0.074^a^±0.002 |
| Psicose-meto-5TMS | 0.032^c^±0.001 | 0.094^a^±0.008 | 0.082^a^±0.002 | 0.076^b^±0.020 | 0.083^a^±0.003 | 0.075^b^±0.003 |
| Fructose-meto-5TMS | 0.015^d^±0.001 | 0.061^a^±0.003 | 0.052^b^±0.001 | 0.049^c^±0.012 | 0.061^a^±0.004 | 0.056^b^±0.003 |
| Sorbose-meto-5TMS | 0.015^b^±0.001 | 0.061^a^±0.002 | 0.053^a^±0.002 | 0.051^a^±0.013 | 0.063^a^±0.005 | 0.057^a^±0.004 |
| Allose-meto-5TMS | 0.291^c^±0.054 | 1.169^a^±0.452 | 0.797^b^±0.237 | 0.862^b^±0.162 | 1.498^a^±0.073 | 1.038^a^±0.354 |
| Glucono-1,5-lactone-4TMS | 0.153^d^±0.032 | 0.667^c^±0.027 | 0.530^c^±0.022 | 0.546^c^±0.147 | 0.973^a^±0.120 | 0.859^b^±0.100 |
| Mannose-meto-5TMS | 0.242^d^±0.083 | 1.208^b^±0.379 | 0.859^c^±0.198 | 0.908^c^±0.093 | 1.596^a^±0.104 | 1.157^b^±0.279 |
| Galactose-meto-5TMS | 0.273^e^±0.081 | 1.566^c^±0.158 | 1.089^d^±0.013 | 1.185^d^±0.171 | 1.725^b^±0.098 | 1.821^a^±0.083 |
| Glucose-meto-5TMS | 0.343^d^±0.066 | 1.484^b^±0.365 | 1.007^c^±0.211 | 1.052^c^±0.126 | 1.822^a^±0.107 | 1.432^b^±0.275 |
| Mannitol-6TMS | 0.182^d^±0.028 | 0.909^a^±0.137 | 0.552^c^±0.013 | 0.666^c^±0.039 | 0.814^b^±0.018 | 0.989^a^±0.041 |
| Sorbitol-6TMS | 0.145±0.099 | 0.008±0.000 | 0.205±0.279 | 0.006±0.000 | 0.007±0.001 | 0.357±0.493 |
| Glycerol 3-phosphate-4TMS | 0.027^b^±0.000 | 0.114^a^±0.006 | 0.138^a^±0.007 | 0.127^a^±0.011 | 0.143^a^±0.003 | 0.204^a^±0.017 |
| Galactitol-6TMS | 0.188±0.030 | 0.815±0.000 | 0.555±0.017 | 0.716±0.000 | 0.799±0.000 | 0.948±0.000 |
| Glucose 6-phosphate-meto-6TMS | 0.090^b^±0.065 | 0.382^a^±0.014 | 0.278^a^±0.032 | 0.359^a^±0.026 | 0.318^a^±0.006 | 0.392^a^±0.021 |
| Sucrose-8TMS | 0.035±0.012 | 0.085±0.057 | 0.072±0.045 | 0.046±0.021 | 0.023±0.012 | 0.017±0.001 |
| Trehalose-8TMS | 5.611^b^±2.259 | 10.881^a^±0.572 | 9.052^a^±0.606 | 7.965^a^±0.201 | 9.123^a^±0.262 | 9.740^a^±0.409 |
| Maltose-meto-8TMS | 1.567^b^±0.602 | 2.869^a^±0.150 | 2.405^a^±0.139 | 2.103^a^±0.069 | 2.401^a^±0.060 | 2.553^a^±0.096 |
| Glycerol-3TMS | 0.051^c^±0.011 | 0.085^b^±0.001 | 0.089^b^±0.009 | 0.097^b^±0.005 | 0.212^a^±0.004 | 0.209^a^±0.009 |
| 2-Aminoethanol-3TMS | 0.019^d^±0.001 | 0.169^a^±0.011 | 0.136^c^±0.002 | 0.150^b^±0.000 | 0 | 0 |
| Inositol-6TMS | 0.196^d^±0.041 | 0.383^a^±0.036 | 0.325^b^±0.006 | 0.298^c^±0.033 | 0.333^b^±0.008 | 0.343^b^±0.025 |
| **AMINE GROUPS** |  |  |  |  |  |  |
| Hydroxylamine-3TMS | 0.283±0.035 | 0.320±0.067 | 0.290±0.043 | 0.282±0.034 | 0.297±0.060 | 0.332±0.060 |
| N-Acetylglutamine-3TMS | 0 | 0.012^b^±0.001 | 0.010^b^±0.001 | 0.009^b^±0.002 | 0.016^a^±0.002 | 0.014^a^±0.001 |
| Tryptamine-2TMS | 0 | 0.008±0.001 | 0.007±0.000 | 0.007±0.000 | 0.007±0.000 | 0.007±0.001 |
| Glutamine-3TMS | 0.331^c^±0.140 | 0.700^b^±0.109 | 0.848^a^±0.061 | 0.875^a^±0.152 | 1.162^a^±0.176 | 0.952^a^±0.136 |
| Glucosamine-5TMS | 0.010±0.001 | 0.008±0.001 | 0.007±0.000 | 0.007±0.000 |  |  |
| Octopamine-4TMS |  | 0.018^b^±0.001 | 0.020^b^±0.000 | 0.026^b^±0.005 | 0.189^a^±0.015 | 0.011^c^±0.003 |
| Dopamine-4TMS | 0.013^c^±0.000 | 0.130^a^±0.017 | 0.100^a^±0.005 | 0.052^b^±0.009 | 0.050^b^±0.002 | 0.068^b^±0.008 |
| N-Acetylmannosamine-meto-4TMS | 0.017±0.007 | 0.037±0.006 | 0.037±0.011 | 0.028±0.001 | 0.034±0.014 | 0.027±0.016 |
| Cystamine-nTMS | 0.018^b^±0.000 | 0.023^a^±0.002 | 0.021^a^±0.005 | 0.031^a^±0.007 | 0.019^a^±0.002 | 0.024^a^±0.004 |
| O-Phosphoethanolamine-4TMS | 0.033^b^±0.019 | 0.151^a^±0.013 | 0.138^a^±0.014 | 0.147^a^±0.003 | 0.151^a^±0.006 | 0.146^a^±0.008 |
| Putrescine-4TMS | 4.094±2.141 | 8.156±0.308 | 7.715±0.823 | 8.462±0.501 | 8.567±0.154 | 8.958±0.445 |
| **AMINO GROUPS AND OTHERS** |  |  |  |  |  |  |
| Lysine-4TMS | 0.559^c^±0.099 | 1.282^a^±0.067 | 1.050^a^±0.036 | 1.013^b^±0.042 | 1.133^a^±0.074 | 1.110^a^±0.086 |
| Histidine-3TMS | 2.611^b^±1.149 | 9.379^a^±0.247 | 9.294^a^±0.649 | 9.361^a^±0.530 | 9.768^a^±0.607 | 9.995^a^±0.847 |
| Tyrosine-3TMS | 0.027^c^±0.003 | 0.130^a^±0.019 | 0.080^b^±0.002 | 0.097^b^±0.005 | 0.114^a^±0.002 | 0.140^a^±0.006 |
| Proline-2TMS | 1.996^b^±0.550 | 3.660^a^±0.130 | 3.466^a^±0.130 | 3.184^a^±0.134 | 3.357^a^±0.181 | 3.274^a^±0.225 |
| Glycine-3TMS | 1.868^c^±0.404 | 5.412^b^±0.223 | 5.970^b^±0.086 | 5.536^b^±0.221 | 7.962^a^±0.245 | 5.796^b^±0.392 |
| Uracil-2TMS | 0.014^c^±0.001 | 0.026^b^±0.003 | 0.027^b^±0.001 | 0.028^b^±0.001 | 0.038^a^±0.001 | 0.036^a^±0.001 |
| Serine-3TMS | 3.076^c^±0.764 | 3.630^b^±0.167 | 3.644^b^±0.076 | 3.351^b^±0.110 | 5.293^a^±0.082 | 4.181^a^±0.260 |
| Acetylglycine-2TMS | 0.017^b^±0.004 | 0.049^a^±0.003 | 0.044^a^±0.001 | 0.040^a^±0.003 | 0.050^a^±0.002 | 0.046^a^±0.004 |
| Threonine-3TMS | 0.347^b^±0.076 | 0.931^a^±0.057 | 0.834^a^±0.012 | 0.774^a^±0.047 | 0.956^a^±0.042 | 0.857^a^±0.064 |
| Glyceraldehyde-meto-2TMS |  | 0.018^b^±0.001 | 0.018^b^±0.001 | 0.017^b^±0.000 | 0.022^a^±0.001 | 0.018^b^±0.002 |
| Leucine-2TMS | 0.903^c^±0.114 | 2.655^b^±0.142 | 2.528^b^±0.099 | 2.465^b^±0.221 | 3.110^a^±0.133 | 2.788^b^±0.181 |
| Methionine-2TMS | 0.030^b^±0.003 | 0.068^a^±0.003 | 0.079^a^±0.003 | 0.075^a^±0.001 | 0 | 0 |
| 4-Hydroxyproline-3TMS | 0.139^a^±0.016 | 0.385^b^±0.020 | 0.364^b^±0.012 | 0.348^b^±0.013 | 0.456^a^±0.010 | 0.417^a^±0.027 |
| 5-Oxoproline-2TMS | 0.012±0.004 | 0.030±0.014 | 0.039±0.001 | 0.034±0.002 | 0.042±0.002 | 0.042±0.000 |
| Cysteine-3TMS | 0.052^c^±0.009c | 0.120^a^±0.008 | 0.107^b^±0.003 | 0.121^a^±0.007 | 0.137^a^±0.008 | 0.010^b^±0.001 |
| Ornithine-3TMS | 0.039^c^±0.003 | 0.216^b^±0.019 | 0.225^b^±0.015 | 0.236^b^±0.018 | 0.287^a^±0.034 | 0.257^b^±0.022 |
| Phenylalanine-2TMS | 0.492^a^±0.054 | 0.043^c^±0.009 | 0.047^c^±0.004 | 0.055^b^±0.004 | 0.058^b^±0.006 | 0.055^b^±0.004 |
| Xanthine-3TMS |  | 0.023^b^±0.002 | 0.021^b^±0.000 | 0.023^b^±0.000 | 0.029^a^±0.001 | 0.028^a^±0.000 |
| Guanine-3TMS |  | 0.012^c^±0.001 | 0.015^c^±0.002 | 0.019^c^±0.002 | 0.029^a^±0.001 | 0.022^b^±0.001 |
| Kynurenine-3TMS | 0.030^d^±0.011 | 0.102^a^±0.007 | 0.065^c^±0.006 | 0.090^b^±0.008 | 0.063^c^±0.004 | 0.054±0.003 |
| Tryptophan-3TMS | 0.398^c^±0.096 | 2.055^b^±0.116 | 2.193^b^±0.092b | 2.247^b^±0.191 | 2.848^a^±0.186 | 2.313^b^±0.136 |
| Spermidine-5TMS | 0.018^b^±0.000 | 0.023^a^±0.002 | 0.022^a^±0.006a | 0.032^a^±0.008 | 0.019^a^±0.001 | 0.024^a^±0.004 |
| 2'-Deoxyuridine-3TMS |  | 0.017^b^±0.002 | 0.013^c^±0.001 | 0.016^b^±0.001 | 0.022^a^±0.001 | 0.022^a^±0.002 |
| Uridine-4TMS |  | 0.029^b^±0.003 | 0.021^b^±0.002 | 0.026^b^±0.002 | 0.038^a^±0.003 | 0.037^a^±0.003 |
| Uridine-3TMS | 0.009^d^±0.001 | 0.012^c^±0.001 | 0.012^c^±0.001 | 0.039^a^±0.024 | 0.024^b^±0.015 | 0.013^c^±0.001 |
| Inosine-4TMS |  | 0.036^a^±0.002 | 0.027^b^±0.004 | 0.026^b^±0.006 | 0.024^b^±0.001 | 0.036^a^±0.003 |
| Adenosine-4TMS | 0.012^b^±0.000 | 0.046^a^±0.004 | 0.030^a^±0.004 | 0.046^a^±0.001 | 0.034^a^±0.006 | 0.038^a^±0.003 |
| Cytidine-4TMS | 0.022^b^±0.001 | 0.138^a^±0.004 | 0.107^a^±0.006 | 0.128^a^±0.004 | 0.094^a^±0.010 | 0.110^a^±0.005 |
| Monostearin-2TMS | 0.510±0.093 | 0.487±0.015 | 0.418±0.021 | 0.433±0.056 | 0.490±0.032 | 0.538±0.076 |
| Ornithine-d7-4TMS | 0.134±0.089 | 0.021±0.002 | 0.022±0.002 | 0.021±0.001 | 0.018±0.001 | 0.019±0.001 |
| Arginine-3TMS | 0.102±0.002 | 0.015±0.001 | 0.015±0.000 | 0.016±0.001 | 0.015±0.000 | 0.013±0.001 |
